# Outer membrane vesicle-associated lipase FtlA enhances cellular invasion and virulence in *Francisella tularensis* LVS

**DOI:** 10.1038/emi.2017.53

**Published:** 2017-07-26

**Authors:** Fei Chen, Guolin Cui, Shuxia Wang, Manoj Kumar Mohan Nair, Lihong He, Xinyi Qi, Xiangmin Han, Hanqi Zhang, Jing-Ren Zhang, Jingliang Su

**Affiliations:** 1 Key Laboratory of Animal Epidemiology and Zoonosis, Ministry of Agriculture, College of Veterinary Medicine, China Agricultural University, Beijing 100193, China; 2 Beckman Coulter, 36 Cherry Hill Drive, Danvers, MA 01923, USA; 3 Center for Infectious Disease Research, School of Medicine, Tsinghua University, Beijing 100084, China

**Keywords:** *Francisella tularensis*, FTL_0430, FtlA, intracellular infection, lipase, outer membrane vesicle, virulence

## Abstract

*Francisella tularensis* is a highly infectious intracellular pathogen that infects a wide range of host species and causes fatal pneumonic tularemia in humans. *ftlA* was identified as a potential virulence determinant of the *F. tularensis* live vaccine strain (LVS) in our previous transposon screen, but its function remained undefined. Here, we show that an unmarked deletion mutant of *ftlA* was avirulent in a pneumonia mouse model with a severely impaired capacity to infect host cells. Consistent with its sequence homology with GDSL lipase/esterase family proteins, the FtlA protein displayed lipolytic activity in both *E. coli* and *F. tularensis* with a preference for relatively short carbon-chain substrates. FtlA thus represents the first *F. tularensis* lipase to promote bacterial infection of host cells and *in vivo* fitness. As a cytoplasmic protein, we found that FtlA was secreted into the extracellular environment as a component of outer membrane vesicles (OMVs). Further confocal microscopy analysis revealed that the FtlA-containing OMVs isolated from *F. tularensis* LVS attached to the host cell membrane. Finally, the OMV-associated FtlA protein complemented the genetic deficiency of the Δ*ftlA* mutant in terms of host cell infection when OMVs purified from the parent strain were co-incubated with the mutant bacteria. These lines of evidence strongly suggest that the FtlA lipase promotes *F. tularensis* adhesion and internalization by modifying bacterial and/or host molecule(s) when it is secreted as a component of OMVs.

## INTRODUCTION

*Francisella tularensis* is a Gram-negative bacterium that causes zoonotic tularemia. It infects a wide range of hosts, including amoebae, insects, fish, amphibians, birds, small mammals, lagomorphs and primates.^1, 2^ Humans are accidental hosts that are infected by multiple routes, such as the bites of arthropod vectors, contact with infected animals, and inhalation of aerosolized bacteria.^3^ *Francisella* is divided into four subspecies: *tularensis* (type A), *holarctica* (type B), *mediasiatica* and *novicida.*^4, 5^ The strains of types A and B cause the vast majority of human infections due to their relatively higher virulence.^6^ *F. tularensis* subsp. *tularensis* is classified as a category A biowarfare agent because of its extraordinarily low infectious dose and its ability to be aerosolized easily.^4^ When humans inhale *F. tularensis*, the bacteria invade primarily alveolar macrophages and epithelial cells.^4^ Once intracellular bacteria escape from the phagosome, they replicate in the cytosol until the host cell lyses, thereby allowing them to spread to other cells.^7, 8^ The virulence determinants of *F. tularensis* remain largely undefined.^9^ The known virulence factors include lipopolysaccharide (LPS),^10, 11^ MglA/MglB,^12^ AcpA,^13^ DsbB,^14^ FipB,^15^ FipA,^16^ FmvB,^17^ MsrB,^18^ catalase^19^ and the proteins of the type-VI secretion system encoded by the *Francisella* pathogenicity island (FPI).^20^ Several large genetic screens in animal infection models have also revealed many genes that may be involved in *F. tularensis* pathogenesis.^21, 22, 23, 24^

Lipases (EC 3.1.1.3) and esterases (EC 3.1.1.1), collectively known as lipolytic enzymes, are widely found in both prokaryotic and eukaryotic organisms.^25, 26^ These enzymes are characterized by their ability to catalyze the hydrolysis of ester bonds from diverse substrates, as well as the reverse reactions;^27^ thus, they have attracted enormous attention because of their applications as biocatalysts. Apart from their potential industrial uses, lipolytic enzymes have shown to contribute to the fitness and pathogenesis of animal and plant pathogens.^26, 28, 29^ Pathogenic bacteria synthesize and secrete lipolytic enzymes to fulfill a variety of functions, including nutrient acquisition, colonization, invasion of host cells and modulation of host defense. The lipolytic enzymes produced by gut pathogens, such as *Helicobacter pylori* and *Campylobacter pylori*, are able to degrade mucosal lipids and disrupt phospholipids on the epithelial surface, thereby altering mucosal integrity and promoting bacterial colonization and invasion.^30, 31, 32^

Multiple lipolytic enzymes have also been shown to promote the pathogenesis of intracellular pathogens.^29^ SseJ of *Salmonella enterica* serovar Typhimurium, a member of the GDSL lipase family, possesses the activities of phospholipase A, deacylase and glycerophospholipid: cholesterol acyltransferase.^33, 34, 35^ It interacts with and modifies the host membrane to promote bacterial intracellular survival after being secreted from the *Salmonella*-containing vacuole into the host cell cytoplasm.^36^ *Legionella pneumophila* produces at least 15 cell-associated or secreted lipolytic enzymes.^37^ These enzymes are involved in lipid metabolism, lipid degradation and pulmonary surfactant cleavage^38^ and promote nutrient release and bacterial fitness. *In silico* analyses have revealed the presence of ~30 putative genes encoding lipolytic enzymes in *Mycobacterium tuberculosis.*^39^ Some of the gene products have been demonstrated to degrade lipids for bacterial nutrient acquisition,^40^ induce immune responses, and contribute to cytolytic activity.^41^

Outer membrane vesicles (OMVs) represent spherical bi-layer structures naturally spun off from the outer membrane of Gram-negative bacteria. These structures are not only produced under *in vitro* growth conditions^42^ but are also detected in tissues and serum of infected host.^43, 44^ Multiple functions have been attributed to OMVs, such as nutrient acquisition, inter-species communication and biofilm formation.^45^ Moreover, OMVs have been implicated in promoting bacterial pathogenesis by stabilizing toxins,^46, 47^ promoting bacterial adhesion to host cells,^48^ and regulating the adaptive immunity of the host.^49^ Although the OMVs are mostly composed of outer membrane and periplasmic proteins, some cytosolic and inner membrane proteins, RNA, DNA, peptidoglycan and lipopolysaccharide (LPS) are also present in the OMVs of certain bacteria.^45, 50^ It has been well documented that the proteins in the OMVs are a result of selective recruitment because certain low-abundance molecules are highly enriched in the OMVs.^42^ As an example, OMVs from *Pseudomonas aeruginosa* predominantly consist of B-band LPS.^51^ A recent study has identified the DegP protease as a key regulator of protein composition in the OMVs of *Vibrio cholera*.^52^ However, the mechanisms governing the biogenesis of bacterial OMVs remain obscure.^53, 54, 55^ *F. tularensis* subsp. *novicida* and *philomiragia* (referred to hereafter as *F. novicida* and *F. philomiragia*, respectively) are able to produce OMVs, which contain hundreds of proteins.^56, 57^ However, the functional impact of the OMVs on *Francisella* pathogenesis is unclear.

In our previous *in vivo* screen of virulence factors by signature-tagged mutagenesis (STM),^22^ we identified 95 genes in *F. tularensis* live vaccine strain (LVS) that are associated with lung infection. Among these are the genes in the *capBCA* and protease loci, which were subsequently confirmed to be necessary for *F. tularensis* LVS growth in macrophages and mouse organs.^22, 58^ The contribution of many other genes to *Francisella* pathogenesis remains to be defined. This study reports our analysis of *FTL_0430* (named *ftlA*), one of the 95 genes identified as potential virulence factors of *F. tularensis* LVS in the same study.^22^ The unmarked deletion mutant of *ftlA* was significantly impaired in intracellular growth and fitness in a mouse lung infection model. We further showed that *ftlA* encodes a lipase with a preference for short-carbon substrates. Finally, our data also revealed that FtlA is discharged as a component of *Francisella* OMVs. The OMA-associated FtlA is able to enhance *F. tularensis* internalization in cultured macrophages.

## MATERIALS AND METHODS

### Bacterial strains and growth conditions

The *F. tularensis* subsp. *holarctica* LVS was kindly provided by Karen Elkins. LVS and its derivatives were cultured in Mueller-Hinton broth (MHB) or brain-heart infusion (BHI) broth supplemented with 0.1% glucose and 1% IsoVitaleX in a shaker at 37 °C, or on Mueller-Hinton II chocolate agar (MHA) plates containing the same supplements. When necessary, kanamycin (10 μg/mL) and hygromycin (200 μg/mL) were added to the medium for selection purposes. *Escherichia coli* strains were grown in Luria-Bertani (LB) broth or on agar plates. Antibiotics were added as previously described.^22^

### Antibody generation and Western blotting

Antisera against *Francisella* FtlA, FopA and IglC were prepared with recombinant proteins essentially as described.^58^ FtlA was expressed as GST fusion protein (GST-FtlA) by amplifying the *ftlA* open reading frame from LVS genomic DNA with the primer pair Pr1651/Pr1652 (Supplementary Table S1), and cloning it into the plasmid pGEX-2T. *fopA* and *iglC* were similarly cloned into plasmid pET-32a (Novagen) with primer pair Pr2008/Pr2009 and Pr2006/Pr2007 (Supplementary Table S1), respectively. The resulting plasmids were transformed into *E. coli* BL21(DE3) and processed to produce proteins. Recombinant proteins were purified by affinity chromatography with glutathione-Sepharose (GST-FtlA) and Ni-Agarose (His-FopA and His-IglC) according to manufacturers’ instructions. To generate antiserum against FtlA, 500 μg of purified GST-FtlA in 500 μL PBS was emulsified with an equal volume of complete Freund’s adjuvant (Sigma-Aldrich, St Louis, MO, USA) and injected subcutaneously into New Zealand White rabbits. The rabbits were boosted twice at 2-week intervals with a similar amount of protein emulsified with incomplete Freund’s adjuvant (Sigma-Aldrich). Three weeks after the third immunization, the rabbits were anesthetized and cardiac punctured to collect blood. Serum was removed and stored at −80 °C for use. Antisera against FopA and IglC were generated in a similar manner in BALB/c mice (6–8 weeks old) using the purified His-tagged FopA and IglC, respectively.

Western blotting was performed as described.^58^ Antisera against FtlA, FopA and IglC were used at dilutions of 1:1000, 1:5000 and 1:1000, respectively. Horseradish peroxide (HRP)-conjugated second antibodies (ZSGB-BIO, Beijing, China) were used at dilutions of 1:5000.

### Construction of *F. tularensis ftlA* deletion mutant and complementation plasmid

A *ftlA* deletion mutant was constructed by counterselection as described.^22^ The upstream and downstream fragments of *ftlA* were amplified using LVS genomic DNA and the primer pairs Pr1089/Pr1090 and Pr1091/Pr1092 (Supplementary Table S1), respectively, and joined by overlapping PCR. The fusion was digested with *Msp*I and *Dra*I, and cloned into the *Cla*I/*Sma*I sites in the pBlueScript II SK (−) plasmid to obtain pBS::Δ*ftlA*, resulting in plasmid pST1146. The pST1146 insert was sub-cloned into the *Apa*l/*Bam*HI sites of the suicide vector pMP590^59^ to create plasmid pST1147. This plasmid was electroporated into strain LVS to generate an unmarked deletion in *ftlA* by counterselection with 5% sucrose. The *ftlA* deletion in the resulting strain ST1705 was confirmed by DNA sequencing and Western blotting using rabbit antiserum against FtlA. The growth kinetics of the mutant in broth cultures were tested by optical density analysis.

To construct *ftlA* complemented strain, the full-length gene was amplified using primer pair Pr1601/Pr1602 (Supplementary Table S1) and cloned into the *Nde*I site of the pMP633 shuttle vector, in which the ATG of the *Nde*I site corresponds to the start codon of the hygromycin gene in pMP633.^59^ The resulting plasmid pST1729 was verified by DNA sequencing and electroporated into strain ST1705. Transformants were selected on MHA plates containing hygromycin (200 μg/mL). One of the resulting transformants, ST1738, was used for further characterization.

### Quantitative RT-PCR (qRT-PCR)

*F. tularensis* LVS, Δ*ftlA* mutant (ST1705), and *in trans* complemented strain (ST1738) were individually cultured in MHB to mid-log phase (*OD*_600_=1.0). Bacterial RNA was isolated from cultures using TRIzol reagent (Ambion, Austin, TX, USA) and subjected to DNase I (NEB, England, Ipswich) treatment to eliminate genomic DNA contamination.

To determine whether the five genes (*FTL_0427* to *FTL_0431*) are cotranscribed, the junction fragments between these genes were amplified by RT-PCR. The primer pairs covering the junction of adjacent genes were designed using the Primer express software (Primer 3 Plus) and are listed in Supplementary Table S1. The intergenic cDNAs were synthesized with the LVS RNA extract using a Reverse Transcription System (Promega, Madison, WI, USA) and primers Pr2031, Pr2033, Pr2035 and Pr2037, respectively. The junction PCR products were amplified using the synthesized cDNAs and primer pairs (Supplementary Table S1).

To determine whether the deletion of *ftlA* (*FTL_0430*) affects the transcription of its adjacent genes, transcription of the four genes (*FTL_0427*, *FTL_0428*, *FTL_0429* and *FTL_0431*) in the operon was analyzed by qRT-PCR in a two-step reaction. First, cDNA was synthesized using the Reverse Transcription System with the above-described bacterial RNA and random primers according to the manufacturer’s guidelines. qPCR assays were performed with SuperReal PreMix Plus (SYBR Green) (TIANGEN, Beijing, China) using the Light Cycler 480 system (Roche, Basel, Switzerland) with a program of 40 cycles, with each cycle consisting of 95 °C for 10 s and 60 °C for 20 s. The primers used for qRT-PCR are listed in Supplementary Table S1. All reactions were performed in triplicate with three independent RNA preparations. The transcript levels of the target genes were normalized to the levels of 16S rRNA. The results were analyzed using the 2^−ΔΔCT^ method.^60^ Statistical significance was determined using Student’s *t*-test.

### Detection of lipase activity

To detect the lipase activity of FtlA, the *ftlA* gene was amplified using primer pair Pr2000/Pr2001 (Supplementary Table S1) and cloned into pET-32a. The plasmid (pST2000) was transformed into *E. coli* BL21 (DE3) to generate strain ST2000. As a control, the active site serine (Ser_13_) within the conserved lipase motif of FtlA was mutated by site-directed mutagenesis. The serine of the FtlA was converted to alanine (TCT to GCT) using the primer pair Pr2002/Pr2003 (Supplementary Table S1). The *E. coli* strain ST2001 expressing mutated FtlA (FtlA^S13A^) was constructed as described above.

Lipase activity in bacterial cells was detected by a plate assay using tributyrin as a lipase indicator essentially as described.^61^ Briefly, the lipase reporter plates were prepared as follows: 1 mL of tributyrin (Sigma-Aldrich) was added to 100 mL of LB agar for *E. coli*; the mixture was emulsified by sonication immediately before autoclaving. When the temperature of the medium was stabilized to 55 °C after autoclaving, ampicillin and isopropyl β-D-1-thiogalactopyranoside were added to final concentrations of 100 μg/mL and 1 mM, respectively. The mixture was used to prepare the reporter plates for lipase activity. Lipase activity was indicated by the formation of a clear zone after 10 μL of mid-log bacterial cultures (~0.6 *OD*_600_) was spotted and incubated at 37 °C for 10 days. For the lipolytic assay of *F. tularensis* LVS and its isogenic derivatives, 2 × Chamberlain’s chemical defined medium (CCDM) was prepared and sterilized by filtration.^62^ The medium was mixed with an equal volume of agarose emulsified with 2% tributyrin and dispensed onto the plates. Bacterial cultures (~0.8 *OD*_600_) were spotted and incubated at 37 °C with 5% CO_2_ for seven days.

### Protease accessibility

Protease accessibility to bacterial proteins in the cellular or OMV context was performed essentially as described.^63^ Briefly, late-log phase bacteria (~2 × 10^9^ CFU/mL) were pelleted by centrifugation at 8000*g* for 10 min at 4 °C and gently resuspended. Proteinase K (Merck, Darmstadt, Germany) in proteolysis buffer (10 mM Tris–HCl, pH 8.0, 5 mM CaCl_2_) was added to final concentrations of 250–1000 μg/mL, or 0.1% Triton X-100 and proteinase K (to a final concentration of 250 μg/mL) were added. After incubation at 37 °C for 1 h, the reaction was stopped by the addition of a 0.5 mM protease inhibitor cocktail (Sigma-Aldrich). As a negative control, proteolysis buffer alone was added to the cell suspension. Cells were pelleted by centrifugation (8000*g* for 10 min at 4 °C), washed twice with PBS containing the 0.5 mM protease inhibitor cocktail, and resuspended for immunoblot analysis. The target proteins were detected with rabbit antiserum against FtlA or FopA by Western blotting.

Protease accessibility of FtlA in freshly purified OMVs was assessed with proteinase K alone or with SDS (0.02%) for 30 min at room temperature as described.^57^ Reactions were terminated by the addition of a 0.5 mM protease inhibitor cocktail. FtlA in the samples was detected with anti-FtlA serum by Western blotting.

### Subcellular fractionation

Subcellular fractionation of *F. tularensis* proteins was carried out as described.^57^ *F. tularensis* LVS was cultured to the exponential phase in MHB, pelleted by centrifugation (8000*g* for 10 min at 4 °C), and resuspended in lysis buffer (10 mM Tris, pH 7.5, 150 mM NaCl). Bacteria were disrupted by sonication, and unbroken cells were removed by centrifugation at 10 000 *g* for 20 min at 4 °C. The lysate was filtered with a 0.45-μm pore size filter unit (Merck) and subjected to ultracentrifugation (Beckman Optima L-100XP, rotor Type 70 Ti) for 1 h at 100 000 *g* at 4 °C to pellet the membrane components. The supernatant (soluble protein fraction) was removed, and the pellet was resuspended in 1% sarkosyl (Sigma-Aldrich). The sarkosyl-soluble fraction (inner membrane) and sarkosyl-insoluble fraction (outer membrane) were further separated by ultracentrifugation for 1 h at 100 000 *g* at 4 °C. The protoplasts and the periplasmic content were separated by an osmotic shock treatment as previously described.^64^ Briefly, bacteria were pelleted by centrifugation (8000*g*, 10 min at 4 °C), suspended and washed twice with cold Tris–HCl buffer (20 mM, pH 7.5), and then suspended in a hypertonic solution containing 20 mM Tris–HCl (pH 7.5), 20% sucrose, and 0.5 mM ethylenediaminetetraacetic acid. Lysozyme was added to a final concentration of 200 μg/mL. After incubation on ice for 30 min, the cells were pelleted at 12 000 *g*, 4 °C for 10 min. The supernatant was labeled the periplasmic fraction. All the fractions were separated by SDS–polyacrylamide gel electrophoresis (SDS–PAGE) and analyzed by Western blotting.

### Purification of outer membrane vesicles

OMVs were isolated from *F. tularensis* LVS and its derivatives virtually as described.^57^ Briefly, *F. tularensis* LVS and isogenic Δ*ftlA* mutant were cultured in BHI broth to the late exponential phase. Bacteria were removed by centrifugation (8000*g*, 10 min), and the supernatant was filtered using a filter with a 0.45-μm pore. The resulting solution was initially concentrated with an Amicon Ultra-15 filtration unit (Merck), and then subjected to ultracentrifugation at 100 000 *g* for 1 h to pellet the vesicles. The pelleted OMVs were resuspended in buffer (20 mM HEPES (pH 7.5), 0.05% sodium azide). For the gradient density separation, 0.5 mL of the OMV suspension was mixed with 1.5 mL of OptiPrep (60% iodixanol, Axis-Shield) to adjust the OptiPrep concentration to 40% (w/v) before being placed at the bottom of the ultracentrifuge tube. OptiPrep gradients of 35%, 30%, 25%, 20%, 15% and 0% were layered over the vesicle fractions and subjected to centrifugation at 100 000 *g* for 16 h at 4 °C in a swinging-bucket rotor (Beckman Optima L-100XP, rotor Type SW 41Ti). Equal fractions were collected from the tops of the tubes. A portion of each fraction was probed by Western blotting with FtlA antiserum. Simultaneously, 10 μL of each fraction was spotted onto LB plates containing tributyrin indicator for lipase activity detection as described above. Fractions containing FtlA were diluted and recovered by ultracentrifugation (100 000 *g*, 1 h at 4 °C). Pellets were resuspended in 20 mM HEPES (pH 7.5) for morphological and biochemical characterization.

### Detection of OMV attachment to host cells

Confocal laser scanning microscopy analysis of OMV attachment to RAW264.7 cells was conducted as described,^65^ with some modifications. RAW264.7 cells were seeded on glass coverslips in 24-well-plates. Cell monolayers were treated with methyl-β-cyclodextrin (MβCD) (5 mM) or RPMI 1640 medium alone for 1 h. After treatment, the cells were washed with PBS and incubated with 25 μg of OMVs purified from LVS for 1 h at 37 °C. The cells were then washed three times with PBS and fixed with 4% paraformaldehyde solution (v/v) for 10 min at room temperature. The cells were again washed three times with PBS and blocked with 1% bovine serum albumen in PBS (blocking buffer) for 30 min at room temperature. Subsequently, the cells were incubated with rabbit anti-FtlA serum (1:2500) and a monoclonal antibody against caveolin-1 (1:500) (Abcam, UK) in blocking buffer for 1 h and then washed with PBS. DyLight 488-conjugated mouse anti-rabbit IgG (1:500) and DyLight 549-conjugated goat anti-mouse IgG (1:500) (EarthOx, USA) were added and incubated for 30 min. After three washes with PBS, 4′,6-diamidino-2-phenylindole was added to stain the cell nuclei. The coverslips were washed with PBS, mounted on glass slides and sealed with neutral resin. Samples were visualized under a confocal laser scanning microscope (Zeiss LSM 710).

### Transmission electron microscopy

Transmission electron microscopy (TEM) was carried out essentially as described.^56^ OMVs isolated from *F. tularensis* LVS and its derivatives were mounted on carbon-coated copper grids, incubated for 10 min, and washed twice with water. Uranyl acetate (2%, pH 4.0) was applied to the grids for negative staining. The stained grids were subjected to TEM analysis with a JEM-1400 microscope at 80 kV.

### Host cell infection

Infection of host cells by *F. tularensis* LVS and its derivatives was assessed in murine bone marrow-derived macrophages (BMDMs) or mouse macrophage-like RAW264.7 or human lung epithelial A549 cell lines in 24-well cell culture plates as described previously.^56^ RAW264.7 and A549 cells were maintained in RPMI 1640 medium supplemented with 10% fetal bovine serum at 37 °C with 5% CO_2_. BMDMs were isolated from the femurs of 6–8-week-old female BALB/c mice and differentiated into macrophages in RPMI 1640 medium at 37 °C with 5% CO_2_ for five days as previously described.^66^ Cell monolayers were washed with PBS, and bacterial suspensions were added at the indicated multiplicity of infection (MOI). After 1 h of incubation at 37 °C, the cells were washed three times with PBS, and extracellular bacteria were killed by incubation in RPMI 1640 medium containing gentamicin (50 μg/mL) for 1 h at 37 °C. After the residual gentamicin was removed by extensive rinsing, the monolayers were lysed to enumerate the viable intracellular bacteria (or colony forming units—CFU) by plating the lysates on MHA plates.

To determine bacterial adhesion, RAW264.7 cells were pre-treated with cytochalasin D (Sigma-Aldrich) at 1 μg/mL for 1 h to disrupt actin polymerization and block the internalization of bacteria as described.^67^ The cells were then infected with *F. tularensis* LVS or its derivatives. After incubation at 37 °C for 2 h, the infected cells were lysed to enumerate the adherent bacteria (CFU).

The impact of OMVs on the ability of *F. tularensis* LVS to infect host cells was tested as described.^65^ Monolayers of RAW264.7 cells in a 24-well-plate were infected with *F. tularensis* LVS or its isogenic mutant at an MOI of 500. Simultaneously, OMVs purified from the supernatants of the LVS or the Δ*ftlA* mutant were added to final concentrations of 10–100 μg/mL. After incubation at 37 °C for 1 h, the extracellular bacteria were killed by applying gentamicin (50 μg/mL), and viable intracellular bacteria were quantified as described above.

### Mouse infection

Animal infection with *F. tularensis* was conducted as previously described.^22^ Groups of five six-week-old female BALB/c mice (Charles River Laboratory, Beijing, China) were anesthetized with Zoletil50 (Virbac, Sante Animale, France) by subcutaneous administration, infected intranasally with *F. tularensis* LVS or its derivatives, and monitored daily for mortality for 21 days.

For the *in vivo* growth study, mice were inoculated intranasally with 3 × 10^3^ CFU of *F. tularensis* LVS, or its isogenic derivatives ST1705 or ST1738. To maintainthe antibiotic selection on the *in trans* complementation plasmid under *in vivo* condition, mice infected with ST1738 were administered hygromycin (50 μg/mouse) by gavage every day after infection. The bacterial burdens in the lung, spleen and liver (*n*=5) were quantified at 4 and 7 days after infection as described.^58^ To evaluate the histopathological changes caused by the deletion mutant, three mice in each group were euthanized on day 7 post-infection. Lungs were collected and fixed with 10% buffered formalin, processed using standard histological methods, and the sections were stained with hematoxylin and eosin.

### Ethics statement

Animal experiments were conducted under protocols approved by the China Agricultural University Animal Ethics Committee, in accordance with the guidelines of the Review of Welfare and Ethics of Laboratory Animals approved by the Beijing Municipality Administration Office of Laboratory Animals.

### Statistical analysis

All experiments represent at least three replicates, with each experiment performed in triplicate. Data were analyzed using an unpaired *t*-test. The results of the representative experiments are presented as the mean±sd. Significant differences are defined by *P* values (two tailed) <0.05 (^*^), <0.01 (^**^), <0.001 (^***^).

## RESULTS

### *ftlA* is required for *F. tularensis* LVS virulence in mice

Our previous STM study identified four independently generated mutants of *ftlA* that were significantly attenuated in the lung infection model.^22^ The four STM mutants each carried a transposon insertion in a unique position in the coding region of FtlA (between nucleotides 102–103, 147–148, 227–228 and 734–735). However, the function of FtlA is completely unknown. *ftlA* is the fourth gene in an operon of five genes, which consists of *FTL_0427* (chromosome partition protein A, *parA*), *FTL_0428* (chromosome partition protein B, *parB*), *FTL_0429* (putative class I glutamine amidotransferase), *FTL_0430* (*Francisella tularensis* lipase A, *ftlA*) and *FTL_0431* (putative hydrolase). The genes and their order are highly conserved among the sequenced genomes of *F. tularensis.* The five genes are cotranscribed under *in vitro* culture conditions as the predicted intergenic fragments were produced by RT-PCR amplification using the total RNA of LVS as template (Supplementary Figure S1A). No PCR product was generated in the absence of reverse transcription, which eliminated the genomic contamination. Because the transposon used in our previous study terminates the transcription of the sequence downstream of its insertion site gene,^22^ we first attempted to clarify the possibility of the polar effect associated with these Δ*ftlA* mutants by constructing an unmarked deletion mutant in *F. tularensis* LVS (strain ST1705), in which a 771-bp coding sequence of FtlA was removed (Supplementary Figure S1B). The expression of FtlA in ST1705 was successfully restored by *in trans* complementation, as assessed by Western blotting with rabbit antiserum against FtlA (Supplementary Figure S1C). *In vitro* culture experiments showed that the growth kinetics of ST1705 were indistinguishable from those of the parent strain in CCDM or MHB (Supplementary Figure S1D), indicating that FtlA is not required for normal growth *in vitro*. In addition, transcription of the adjacent genes (*FTL_0427*, *FTL_0428*, *FTL_0429* and *FTL_0431)* was analyzed by qRT-PCR, and no significant changes were observed in the transcript levels of the four genes between LVS and Δ*ftlA* (ST1705) or between LVS and the *in trans* complemented strain (ST1738) (Supplementary Figure S2).

**Figure 1:**
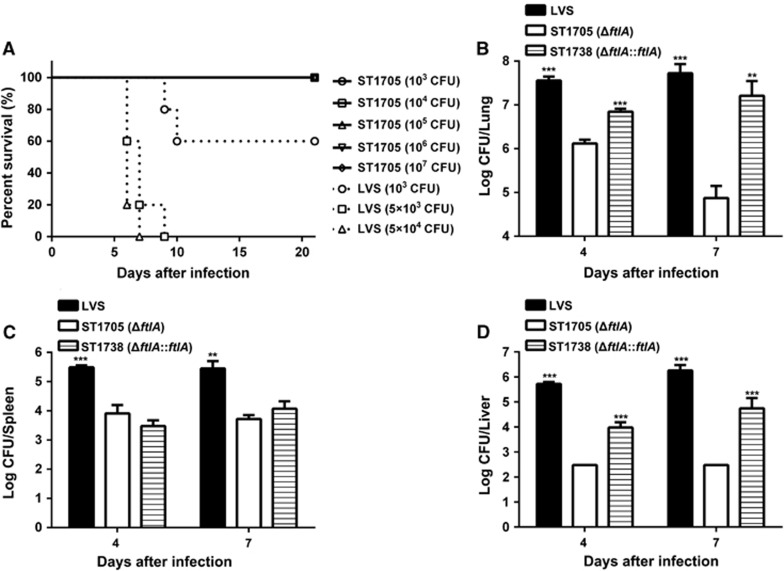
Impact of *ftlA* deletion on the *in vivo* growth and pathogenicity of *F. tularensis* LVS. (**A**) The Δ*ftlA* mutant of LVS is highly attenuated in mice. Groups of BALB/c mice (*n*=5) were intranasally infected with the indicated doses of wild-type LVS and ST1705, and the survival of the mice was plotted over time for 21 days. (**B,C**) The bacterial burden of the Δ*ftlA* mutant ST1705 was significantly lower than that of LVS in mouse organs. BALB/c mice (*n*=5) were infected intranasally with 3 × 10^3^ CFU of wild-type LVS, ST1705 or ST1738, and tissues were collected on days 4 and 7 post-infection.The tissue homogenates were diluted and spread onto MHA plates for CFU analysis. All the values are presented as the mean log CFU±sd. *P* values were calculated using an unpaired *t*-test, comparing the values obtained for ST1705 with those of the parent strain LVS or ST1738.

We next assessed the impact of the *ftlA* deletion on *Francisella* pathogenicity in a mouse lung infection model. While all the mice infected with 5 × 10^3^ CFU of LVS died during an observation period of 21 days, intranasal inoculation with a range of different infection doses (1 × 10^3^ to 10^7^ CFU/mouse) with ST1705 revealed a median lethal dose of ≥10^7^ CFU (Figure 1A). This severe attenuation phenotype of strain ST1705 confirmed our previous finding with the *ftlA* transposon insertion mutants.^22^ Consistent with this conclusion, the mice infected with ST1705 carried dramatically lower bacterial load in the lung, liver and spleen on days 4 and 7 post-infection. There were more than 27-fold and 38-fold differences between the lung (Figure 1B) and spleen (Figure 1C) values in mice infected with ST1705 and LVS on day 4. The Δ*ftlA* mutant was not recovered from the liver at this timepoint (limit of detection, 300 CFU/liver)(Figure 1D). A significant attenuation phenotype of Δ*ftlA* mutant *in vivo* growth was also observed in terms of bacterial load in the lung, liver, and spleen on day 7 post-infection, although the overall levels of bacterial burden detected on day 7 were lower than those obtained on day 4. The *in vivo* growth defect of the Δ*ftlA* mutant was partially rescued by *ftlA in trans* complementation. In comparison with the Δ*ftlA* mutant, the bacterial loads in the lung and liver increased significantly in the mice infected with strain ST1738 (Figures 1B and 1D).

We also compared the pathological changes in the lungs of the mice infected with LVS or ST1705. The histopathological evaluation revealed a consistent correlation between the severity of the pathology and bacterial burden in the lungs on day 7 post-infection. The lungs of the LVS-infected mice exhibited severe pathological damages with sloughed mucosa, erythrocytes and inflammatory cells in the bronchi. The mice infected with LVS displayed marked peribronchial and perivesicular inflammatory cell infiltrations with large numbers of erythrocytes in the alveoli. In contrast, the lungs of the mice infected with ST1705 only showed relatively minor peribronchial and perivesicular inflammatory cell infiltration.This evidence indicates that FtlA is essential for the *in vivo* fitness and virulence of *F. tularensis* LVS in this lung infection model.

### *ftlA* contributes to *Francisella* attachment and entry into host cells

**Figure 2:**
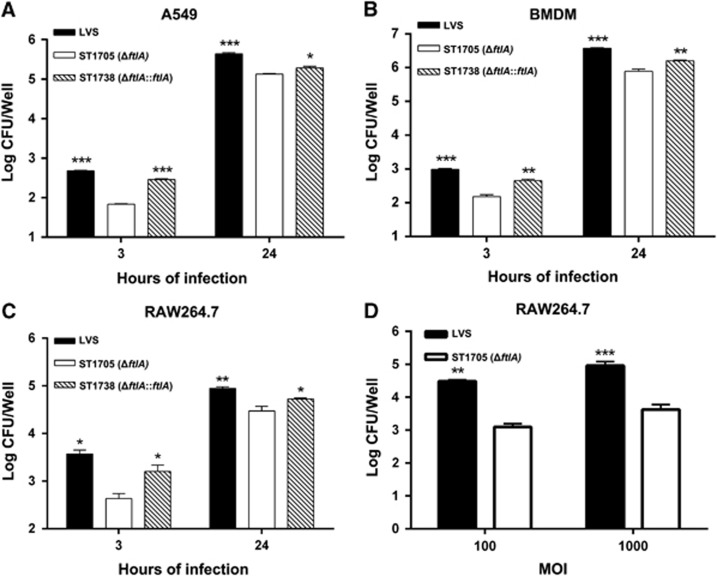
Contribution of FtlA to host cell infection. (**A**–**C**) LVS and mutant derivatives were used to infect human lung epithelium A549 cells, murine bone marrow-derived macrophages or RAW264.7 cells in 24-well plates at a MOI of 100. The cells were lysed at 3 and 24 h post-infection and plated on MHA plates for enumeration of bacterial CFU. (**D**) RAW264.7 cells were treated with cytochalasin D to inhibit internalization and then infected with LVS or Δ*ftlA* mutant at the indicated MOIs. The cells were extensively washed and lysed at 2 h post-infection, and the CFUs of the bacteria that adhered to the RAW264.7 cells were enumerated as above. All the values are presented as the mean log CFU±sd of triplicate samples. *P* values were calculated using an unpaired *t*-test, comparing the value of ST1705 with that of the parent strain LVS or ST1738.

To further explain the severe attenuation of the Δ*ftlA* mutant in the lung infection model, we tested the impact of *ftlA* deletion on *Francisella* attachment and entry into host cells using multiple cell models. The results showed that the Δ*ftlA* mutant exhibited an altered infection profile in all tested cells, compared with wild-type LVS (Figures 2A–C). The A549 cells infected with LVS exhibited seven-fold more intracellular bacteria than those infected with the Δ*ftlA* mutant ST1705. The *in trans* complementation plasmid restored the infectivity of the Δ*ftlA* mutant nearly to the level of the parent strain at 3 h after the initiation of infection (Figure 2A). Despite the relatively smaller scale, the mutant also displayed a significant reduction of the level of intracellular bacteria compared with LVS 24 h after the onset of infection. Consistent distinct patterns of LVS and ST1705 were also observed in the levels of intracellular bacteria in cultures of mouse bone marrow-derived macrophages (Figure 2B) and RAW264.7 mouse macrophages (Figure 2C). This result showed that *ftlA* was necessary for the infectivity of *F. tularensis* LVS at the cellular level. Gradual loss of the shuttle plasmid in the complementation strain due to the absence of antibiotic selection might be responsible for the relatively lower levels of intracellular bacteria compared with the parent strain.

To determine whether the impaired infectivity of ST1705 was due to partial loss of bacterial adhesion to host cells, we performed adhesion experiments with host cells pre-treated with cytochalasin D, an agent that inhibits actin microfilament polymerization, to uncouple adhesion from internalization. A previous study has reported that treatment with cytochalasin D significantly blocks *Francisella* entry into host cells.^67^ The results showed that cell-associated numbers of wild-type LVS increased 24-fold and 22-fold compared with the *ftlA* deletion mutant, at MOIs of 100:1 and 1000:1, respectively (Figure 2D). Taken together, these results demonstrate that *ftlA* contributes to host cell adhesion and entry by *F. tularensis*.

### FtlA is a new lipase in the GDSL esterase/lipase family

**Figure 3:**
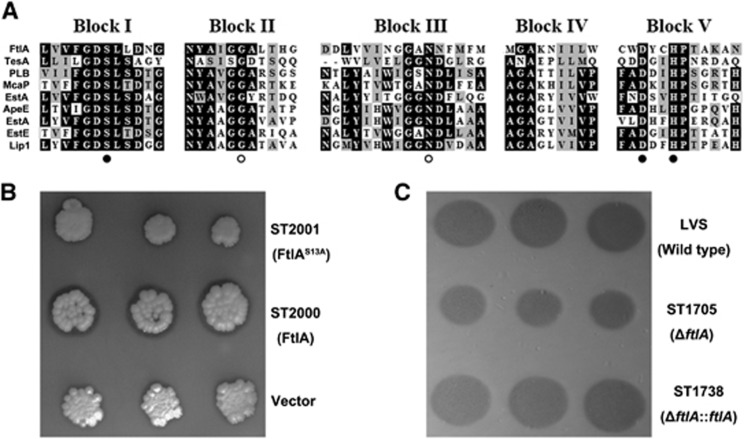
Identification of the lipolytic activity in FtlA. (**A**) Sequence alignment of various bacterial GDSL family serine esterase/lipases depicting five consensus blocks (marked at the top) shared by members using MegAlign of the DNAstar-Lasergene package (version 10). Conserved residues are highlighted with black characters. The residues of the catalytic triad and oxyanion hole are indicated by filled and open circles, respectively. The segments around the five conserved blocks are shown after removal of the junction sequences. FtlA: *Francisella tularensis* (CAJ78870), TesA: *Escherichia coli* (EIQ72915), PLB: *Moraxella bovis* (AAK53448), McaP: *Moraxella catharralis* (AAP97134), EstA: *Pseudomonas aeruginosa* (AAB61674), ApeE: *Salmonella thyphimurium* (AAL19521), *Serratia liquefaciens* EstA (AAO38760), EstE: *Xanthomonas vesicatoria* (AAP49217), Lip1: *Xenorhabdus luminescens* (P40601). (**B**) Lipolytic activity of recombinant *E. coli* on LB agar plates. *E. coli* harboring plasmid pST2000 (ST2000), pST2001 (ST2001) or empty plasmid (vector) were spotted on LB plates containing tributyrin as an indicator. Halo formation was observed around the spots of ST2000 after incubation for ten days at 37 °C. (**C**) The lipolytic activity of *F. tularensis* LVS and its derivatives. LVS, Δ*ftlA* mutant (ST1705) and trans-complemented strain (ST1738) were spotted onto CCDM plates containing tributyrin. The lipase activity in each spot is indicated by the size of the clear zone after incubation for seven days at 37 °C with 5% CO_2_.

*ftlA* consists of 882 base pairs (bps) encoding a protein with 293 amino acids. It is annotated as ‘phospholipase’ in the current version of the LVS genome (NC_007880.1 updated on August 12, 2015) because the protein contains a highly conserved sequence motif of GDSL at the amino (N) terminus, a signature of lipolytic enzymes or lipases in the GDSL family of serine esterases/lipases.^25, 68^ FtlA also possesses five successive conserved blocks (I–V) with four strictly conserved catalytic residues, Ser_13_-Gly_68_-Asn_114_-His_272_, in blocks I, II, III and V, respectively (Figure 3A). The conserved serine (Ser_13_), aspartic acid (Asp_269_) and histidine (His_272_) are predicted to form the catalytic triad of the lipase active site, and the catalytic serine residue is located at the amino terminus.

On the basis of this prediction, we first tested the potential lipolytic activity of FtlA in *E. coli* by generating an expression construct of FtlA in pET-32a. When tested on the plate containing emulsified tributyrin, the *E. coli* strain harboring the FtlA-expressing plasmid (pST2000) showed a clear zone of halo around the colonies (Figure 3B, middle), which is indicative of lipolytic activity as previously reported.^61^ In contrast, no halo was observed around the colonies of the *E. coli* strain containing the empty vector (Figure 3B, bottom). To confirm this result, an S13A *ftlA* mutation construct was generated by substituting its 13th serine residue with an alanine. In agreement with the importance of the serine residue in the lipase activity of the GDSL family proteins, no halo was observed around the colonies of the *E. coli* strain carrying pST2001 (Figure 3B, top). These data demonstrate that recombinant FtlA is a lipolytic enzyme of lipase in *E. coli*.

In a similar manner, we further determined whether FtlA is a functional lipase in *F. tularensis*. Approximately the same CFU of LVS and its isogenic derivatives were spotted onto CCDM agar containing tributyrin. As shown in Figure 3C, the Δ*ftlA* mutant ST1705 produced smaller lipolytic zones around its colonies compared with the wild-type LVS strain after 7 days of incubation. When the *ftlA* gene was introduced into the Δ*ftlA* mutant by *in trans* complementation, the lipolytic zone was restored to the same level as that of the parent strain (Figure 3C). Together with the lipase activity of the recombinant FtlA in *E. coli*, this result showed that FtlA is a functional lipase in *F. tularensis* LVS. Because the *ftlA* gene is highly conserved in *F. tularensis,* we have thus designated it *F. tularensis* lipase A (*ftlA*).

### FtlA is a cytoplasmic protein

**Figure 4:**
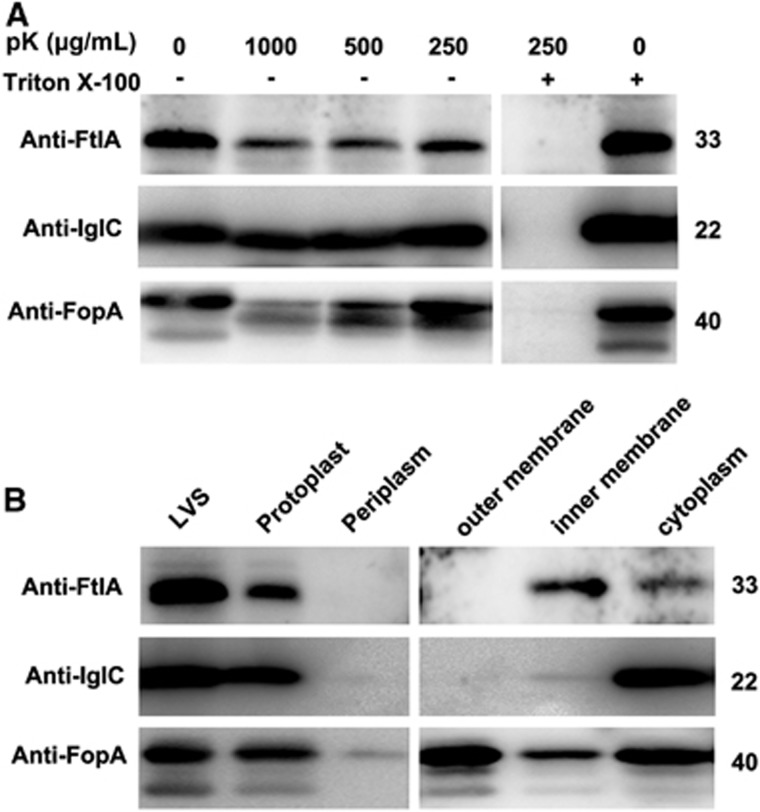
Subcellular localization of FtlA. (**A**) Protease inaccessibility of FtlA. Intact cells of LVS were first incubated with different concentrations of protease K (pK) with or without Triton X-100 permeabilization. FtlA, IglC (cytoplasmic protein control), and FopA (outer membrane protein control) were detected in the cell lysates by Western blotting. (**B**) Distribution of FtlA in subcellular fractions. Subcellular fractionation was performed with intact cells of LVS. FtlA, IglC and FopA were detected in the whole cell lysate (LVS) or subcellular fractions of LVS by Western blotting. The sizes of the proteins are indicated on the right in kDa.

Bioinformatics analysis suggested that FtlA is a cytoplasmic protein since it lacks an apparent signal sequence or transmembrane region. However, this prediction is somewhat paradoxical to the functional contribution of this protein in promoting bacterial adhesion and entry into host cells (Figure 2). Thus, we investigated the cellular localization of the FtlA protein using multiple approaches. First, we determined the potential exposure of FtlA at the cell surface by treating the intact LVS cells with proteinase K, a protease that has been previously used to detect the surface availability of bacterial proteins.^63^ Western blotting revealed that treatment of intact LVS cells with proteinase K resulted in a dose-dependent reduction of FopA, an outer membrane protein of *F. tularensis*,^69^ but the amount of IglC, a cytoplasmic protein of *F. tularensis*,^70^ was not obviously affected by the treatment (Figure 4A), indicating that proteinase K selectively degraded surface-exposed proteins. However, similar treatment with the protease did not show a dose-dependent marginal decrease in FtlA with intact bacterial cells (Figure 4A). This result suggested that FtlA is not surface-exposed, as predicted by bioinformatics analysis of its sequence.

We further determined the precise subcellular localization of FtlA by subcellular fractionation of the LVS proteins into cytoplasmic, inner membrane, periplasmic, and outer membrane fractions. Consistent with its outer membrane localization, FopA was abundantly detected in the outer membrane fraction (Figure 4B). However, FtlA, along with IglC, was detected in the cytoplasmic and inner membrane fractions but not the outer membrane fraction. This finding confirmed our conclusion from the proteinase K treatment experiment (Figure 4A) that FtlA is localized in the bacterial cytosol and absent from the surface of *F. tularensis* LVS.

### FtlA is associated with outer membrane vesicles

**Figure 5:**
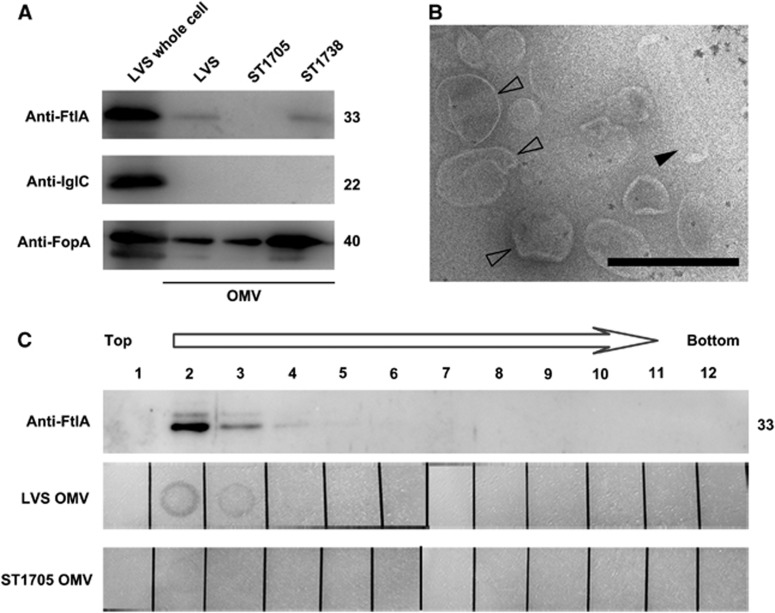
Detection of FtlA in OMVs isolated from *F. tularensis* LVS. (**A**) Detection of FtlA in OMVs by Western blotting. The proteins in the OMVs isolated from LVS and its derivatives were detected with specific antibody against FtlA, IglC, or FopA. (**B**) Electron micrograph of density-gradient purified *F. tularensis* LVS OMVs (negative stained), showing round (open arrow) and rod-shaped (filled arrow) OMVs. Bar=500 nm. (**C**) Analysis of FtlA in the density-gradient-purified OMVs. Crude OMVs extracted from the culture supernatants of LVS or Δ*ftlA* mutant ST1705 were further purified by density-gradient centrifugation. FtlA in the fractions (1–12, top panel) was analyzed by Western blotting as in A. The lipase activity in each OMV fraction (middle and bottom panels) was detected on plates containing tributyrin as in Figure 3. The formation of a halo zone was used as an indicator of lipolytic activity. The sizes of the proteins are indicated on the right in kDa.

A recent proteomics study has reported that the FtlA orthologue of *F. novicida* is associated with OMVs.^57^ To test whether FtlA is released as a component of the OMVs in *F. tularensis* LVS, we isolated OMVs from the exponential-phase supernatants of LVS or its isogenic derivatives by ultracentrifugation. Western blotting detected FtlA and FopA in the OMV preparations of LVS and the *ftlA* complementation strain ST1738 culture supernatant, but not in that of the FtlA-deficient strain ST1705 (Figure 5A). FopA is one of the proteins associated with the OMVs of *F. novicida*.^56^ In addition, FtlA was not detected in the culture supernatant after the OMVs were removed by ultracentrifugation (see Supplementary Material and Supplementary Figure S3). We subsequently characterized the OMVs that were further purified from the initial OMV preparation using density-gradient fractionation. Transmission electron microscopy examination of the purified OMVs revealed spherical and rod-shaped vesicles ranging from 50 to 300 nm in diameter (Figure 5B), resembling the OMVs that are isolated from *F. novicida*^57^ and other bacteria.^71^ In agreement with the results obtained from the initial OMV preparations, FtlA was immunologically detected in density-gradient fractions 2, 3 and 4 of strain LVS (Figure 5C, top panel). This result is consistent with previous findings showing that bacterial OMVs tend to have a relatively low density/high buoyancy.^56^ In accordance with this result, lipase activity was also observed in the same LVS fractions (Figure 5C, middle panel). In contrast, the corresponding OMV fractions of the Δ*ftlA* mutant exhibited much weaker enzymatic activity (Figure 5C, bottom panel). This result correlates well with the lipolytic behavior of the viable bacteria on plates containing tributyrin (Figure 3C).

The OMVs of the LVS spotted on the tributyrin plates were observed to form the halo structure, an indicator of lipase activity, much faster than live bacteria. The haloes of the fresh OMV preparations were visible as early as 3 h and reached the greatest intensity within 48 h after being spotted on the plates. However, it took at least 72 h for the broth cultures of LVS to form visible haloes post-inoculation. Since the fast halo formation did not occur with OMVs isolated from the FtlA-deficient strain ST1705 (Figure 5C), this result suggested that the OMVs contained FtlA protein with a relatively high concentration and/or activity. Since our earlier trial to define the enzymatic activity of FtlA using recombinant FtlA was unsuccessful (not shown), we tested the substrate specificity of FtlA using the OMV preparations (Supplementary Materials). The freshly prepared OMVs of the wild-type LVS hydrolyzed *p*-nitrophenyl butyrate (C_4_) efficiently (Supplementary Figure S4), and a sharp decrease in enzymatic activity was observed for acyl chain lengths greater than ten carbons. In comparison, the hydrolytic activity toward the corresponding *р*-NP ester was significantly lower for the OMVs isolated from the Δ*ftlA* mutant. Taken together, the presence of the FtlA protein in the lower density gradients, as expected for lipid-containing membrane vesicles, and the reduced lipolytic activity of the purified OMVs clearly demonstrate that the *F. holarctica* LVS cytoplasmic protein FtlA is translocated extracellularly as a naturally occurring component of OMVs.

### The OMV-associated FtlA promotes entry of the Δ*ftlA* mutant into host cells

**Figure 6:**
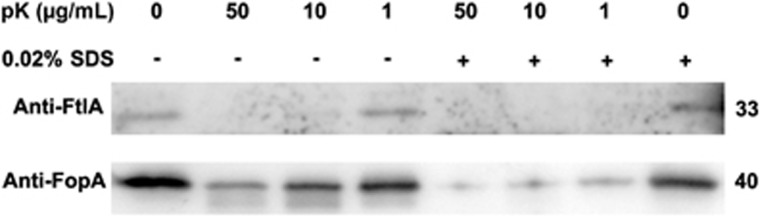
Protease accessibility of OMV-associated FtlA. OMVs purified from *F. tularensis* LVS supernatant were treated with proteinase K in the presence or absence of 0.02% SDS. FtlA and FopA in the samples were probed by Western blotting. The sizes of the proteins are indicated on the right in kDa.

On the basis of the finding that FtlA is required for adhesion and entry into host cells by *F. tularensis* LVS (Figure 2), we determined whether the OMV-associated FtlA is able to interact with host cells. First, we determined the localization of FtlA in the OMVs using the protease accessibility method. Western blotting showed that FtlA became undetectable after the OMVs isolated from LVS were treated with proteinase K for 30 min in the absence of detergent (Figure 6). In contrast, the integral outer membrane protein FopA was only sensitive to proteolysis after the OMV integrity was disrupted by the addition of 0.02% SDS, as reported previously.^57^ This result strongly suggested that FtlA is exposed at the surface of the OMVs.

**Figure 7:**
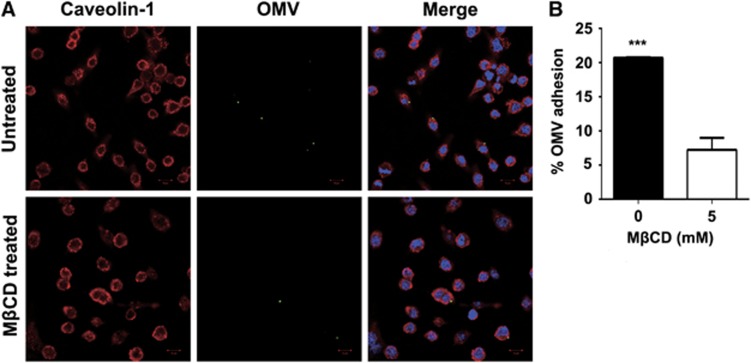
Attachment of FtlA-containing OMVs to host cells. (**A**) Detection of the physical association between FtlA-containing OMVs and host cells. RAW264.7 cells grown on coverslips were incubated with OMVs for 1 h (upper panel) or treated with MβCD prior to OMV incubation (lower panel), and processed for immunostaining of caveolin-1 (host cell membrane) or FtlA (OMVs). The upper panel shows the proximity of the OMVs (green fluorescence) to the host cell membrane, as indicated by caveolin-1 staining (red fluorescence); the lower panel shows the significance of membrane cholesterol in OMV attachment. (**B**) Quantification of the RAW264.7 cell association with *Francisella* OMVs. RAW264.7 cells positively stained with the FtlA antibody (green in A) are expressed as their percentage among the total cells. A total of 600 cells (200 cells/field) were counted. All values are presented as the mean±sd of triplicate samples. *P* values were calculated using an unpaired *t*-test.

We next evaluated the potential attachment of the OMVs to host cells by confocal microscopy using an antibody specific for FtlA (an indicator of *Francisella* OMVs) and caveolin-1 (a marker of the host cell membrane). The immunofluorescence analysis revealed an intimate association between the FtlA-containing OMVs and RAW264.7 cells (Figure 7A). This type of interaction appeared to be mediated by the cholesterol-containing lipid rafts in the host cell membrane because treatment with 5 mM MβCD before the addition of the *Francisella* OMVs significantly reduced OMV attachment (Figure 7B). MβCD selectively extracts membrane cholesterol.^72^

**Figure 8:**
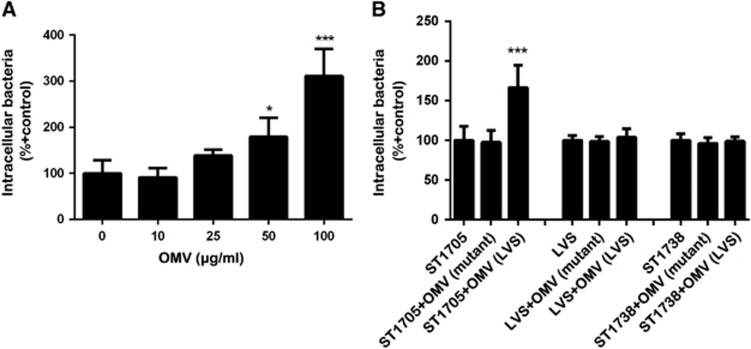
Complementation of the Δ*ftlA* mutant by LVS OMVs and its ability to infect host cells. (**A**) Dose-dependent impact of LVS OMVs on entry of the Δ*ftlA* mutant into host cells. The monolayers of RAW264.7 cells were co-incubated with the Δ*ftlA* mutant ST1705 and OMV preparation from the parent strain at final concentrations of 0–100 μg/mL for 2 h. Internalized bacteria were enumerated by plating the cell lysates on MHA after the extracellular bacteria were removed by gentamicin treatment. (**B**) Contribution of FtlA to OMV-enhanced bacterial entry into host cells. Entry of LVS or ST1705 into RAW264.7 cells were tested in the presence or absence of the OMV preparations from LVS or ST1705 (50 μg/mL) as in A. The values represent the CFU levels of intracellular bacteria±sd of triplicate samples. *P* values were calculated using an unpaired *t*-test.

Finally, we determined whether OMV-associated FtlA is able to complement the deficiency of the Δ*ftlA* mutant in infecting host cells by assessing the intracellular infectivity of strain ST1705 in the presence of OMVs from the parent strain LVS. An initial test with various concentrations of the LVS OMVs (0–100 μg/mL) revealed that the OMVs in the LVS culture enhanced the entry of RAW264.7 cells by the Δ*ftlA* mutant in a dose-dependent manner (Figure 8A). The level of enhancement reached as high as two-fold. To verify the specific role of FtlA in the OMV-enhanced entry of ST1705, various combinations of bacterial strains and OMV preparations were used to perform the cell invasion experiment. While the OMVs from the culture supernatant of the parent strain LVS significantly enhanced the entry of ST1705 into the host cells, similar preparations from ST1705 failed to complement the deficiency of the Δ*ftlA* mutant (Figure 8B). However, the same OMV preparations from the LVS culture did not show a significant impact on cell internalization of strain LVS or the complemented Δ*ftlA* mutant, suggesting that the effect of FtlA on LVS invasion is saturated by expression of the *ftlA* gene in these strains. These results strongly suggest that the OMV-associated FtlA plays an important role in promoting the entry of *F. tularensis* LVS into host cells.

## DISCUSSION

Bacterial factors that contribute to *F. tularensis* adhesion to and invasion of host cells are largely unknown.^67^ This study has revealed that the FtlA lipase plays an important role in *F. tularensis* infection of host cells and *in vivo* fitness based on the following results: (i) genetic deletion of the *ftlA* gene in the genome of *F. tularensis* LVS leads to significant impairment in the ability to infect host cells and complete loss of virulence in the pneumonia mouse model; (ii) FtlA possesses a lipolytic activity in both *E. coli* and *F. tularensis*; (iii) FtlA can be translocated to the extracellular environment as a component of *F. tularensis* OMVs; and (iv) the OMV-associated FtlA promotes bacterial adhesion to, and entry into, host cells. These results strongly suggest that FtlA lipase promotes *F. tularensis* adhesion and internalization by modifying bacterial and/or host molecule(s) when it is secreted as a component of OMVs.

The *ftlA* gene is essential for the virulence of *F. tularensis* LVS. In agreement with the previous finding obtained for the transposon insertion mutants,^22^ the unmarked deletion in the coding sequence of *ftlA* completely abolished the virulence of the parent strain LVS in the pneumonia mouse model, despite the observation that the mutant had no apparent defect in *in vitro* growth. This result is in agreement with the significant reduction of bacterial burden in the lungs, livers and spleens, as well as the dramatic decrease in tissue inflammation in the lungs of the mice infected with the Δ*ftlA* mutant. Consistently, the Δ*ftlA* mutant displayed a significant attenuation of adhesion and entry into host epithelial cells and macrophages, but the deficiency of the same mutant was compensated by *in trans* complementation with the *ftlA* gene.

FtlA is a *Francisella* lipase. The lipolytic activity of FtlA was initially demonstrated, when it was heterologously expressed as a His-tagged recombinant protein in *E. coli*. The *ftlA*-expressing *E. coli* formed a halo around the colonies on the tributyrin-containing agar plates. The halo structure was no longer detectable when the predicted active site serine residue was mutated to alanine. This result confirmed our initial hypothesis that *ftlA* encodes a lipase on the basis of its sequence homology to the members of the GDSL serine esterases/lipases. This conclusion is further supported by the lipase activity of strain LVS, which was diminished in its Δ*ftlA* mutant but resurrected with the *ftlA* complementation plasmid. Finally, the lipase activity was also detected in the OMV preparations of the LVS but not the counterpart of the Δ*ftlA* mutant. We noticed that the lipase activity was more readily detectable in the LVS OMV preparations than the LVS cultures. This phenomenon might be caused by the possibility that the cellular form of the FtlA protein is ‘trapped’ in the cytosol and is not accessible to its substrate(s) unless it is released in the context of the OMVs. While lipolytic activity has been described for the OMVs of *Legionella pneumophila*,^73^ to the best of our knowledge, this study represents the first demonstration that the OMV-associated lipase promotes bacterial infection of host cells.

The present data suggest that FtlA is displayed at the surface of OMVs and thereby becomes accessible to an as yet unknown molecule(s) at the interface of the pathogen-host interaction, although it is localized in the cytosol of bacterial cells. In agreement with its lack of a typical signature sequence for secretion or obvious trans-membrane segments, FtlA was not susceptible to proteolysis in the context of intact bacterial cells. However, it was rapidly degraded when the membrane integrity of the *F. tularensis* cells was compromised by detergent. This finding is in full agreement with the presence of FtlA in the cytoplasmic fraction and inner membrane fractions but not in the outer membrane and periplasmic fractions in the subcellular fractionation experiment. However, our further investigation provided multiple lines of evidence that FtlA is spontaneously released into the extracellular environment as a protein component of OMVs. FtlA was detected, along with the outer membrane protein FopA, in the OMVs extracted from the supernatants of the LVS broth cultures by Western blotting. In contrast, the cytoplasmic protein IglC was undetectable in the OMV preparations, although it was present in the whole cell lysate. There are many examples in which bacterial pathogens use OMVs to selectively secrete virulence factors and thereby facilitate their delivery to host targets.^74^ The heat-labile enterotoxin of enterotoxigenic *E. coli* is found at the surface of OMVs.^46, 47^ Similarly, the Lewis antigens on the lipopolysaccharide molecules of *Helicobacter pylori* are detectable at the surface of OMVs.^75^ Thus, we believe that the FtlA detected in our OMV preparations represents a natural component of the *Francisella* OMVs, instead of protein contamination during the OMV purification. Consistently, the major target(s) of FtlA may be the lipid molecules associated with the surface of bacterial and/or host cells, which facilitate *Francisella* adhesion and entry into host cells. This prediction is consistent with our observation that the FtlA-associated OMVs were detected at the surface of cultured RAW264.7 cells.

Our cell culture infection experiments with the *ftlA* deletion and complementation derivatives of strain LVS strongly suggested that FtlA promoted bacterial adhesion and entry into host cells, with a less important role in the replication of *F. tularensis* within host cells. The *ftlA* mutant showed at least a seven-fold reduction of intracellular bacteria during the initial phase of infection (3 h post-inoculation). This result was reproducible in three cell culture models: primary macrophages (BMDM) and two cell lines (A549—epithelial cell and RAW264.7—macrophage). This impairment was rescued with the *ftlA*-expressing plasmid, thus demonstrating the specific contribution of the *ftlA* gene to the infectivity of strain LVS. In contrast, relatively lower margins of differences (<5-fold) in intracellular bacteria were observed during a later phase of infection (24 h). This result suggests that FtlA is primarily involved in adhesion and internalization rather than intracellular growth. This conclusion is in concordance with the remarked impairment of the *ftlA* mutant (>20-fold) in adhesion to host cells (for example, RAW264.7 cells). Our assignment of FtlA as an important bacterial factor for adhesion and internalization is also consistent with our observation that the OMVs of the parent strain LVS restored the internalization of the *ftlA* mutant in an FtlA-dependent manner since similar OMV preparations from the *ftlA* mutant had no effect. The lack of OMV-mediated enhancement during co-incubation with LVS or the complemented *ftlA* mutant might reflect functional saturation of endogenous FtlA in these strains.

It is tempting to postulate that the FtlA lipase promotes *Francisella* infection of host cells by enzymatic modification of a lipid factor(s)/structure(s) exposed at the surface of either the host or the bacterial cells. There are numerous possibilities that FtlA-mediated modification would facilitate *Francisella* infection of host cells, such as enable the ligand-receptor interaction by modifying either the ligand or the receptor, or by changing the physical properties at the interface between the bacterial and host cells by increasing the leakiness of the host cell/tissue barrier to facilitate bacterial adhesion or entry. The former hypothesis is supported by the increase in bacterial adhesion and entry into host cells by the *ftlA* mutant in the presence of FtlA-containing OMVs. There is growing evidence that bacterial phospholipase-induced lipid-raft reorganization leads to protein clustering and increased local receptor concentrations, which in turn led to enhanced bacterial adhesion and subsequent internalization.^76^ The promotion of *Francisella* infection by the OMV-associated FtlA lipase is reminiscent of a recent report in which the serine proteases in the OMVs of *Campylobacter jejuni*, a food-borne pathogen, enhanced bacterial adhesion to and invasion of human colon epithelial cells via the proteolytic cleavage of E-cadherin and occlusion of two major components of tight junctions.^65^

Taken together with our previous study,^22^ this study provides further evidence supporting FtlA lipase as an important virulence factor of *F. tularensis* LVS and likely other *F. tularensis* subspecies due to the high conservation of the *ftlA* locus in this species. The secretion of FtlA via OMVs represents a novel mechanism of the *F. tularensis* interaction with host cells during the process of pathogenesis because tube-like OMV were produced in *F. novicida* during macrophage infection.^57^ Further characterization of FtlA, in terms of its target molecule(s), mode of recruitment during OMV biogenesis, and impact in type A strains of *F. tularensis*, will be highly desirable for a full understanding of *F. tularensis* virulence and biogenesis of bacterial OMVs.

## References

[bib1] Oyston PC, Sjostedt A, Titball RW. Tularaemia: bioterrorism defence renews interest in *Francisella tularensis*. Nat Rev Microbiol 2004; 2: 967–978. 15550942 10.1038/nrmicro1045

[bib2] Hestvik G, Warns-Petit E, Smith LA et al. The status of tularemia in Europe in a one-health context: a review. Epidemiol Infect 2014; 143: 2137. 25266682 10.1017/S0950268814002398 PMC9506979

[bib3] Sjöstedt A. Tularemia: history, epidemiology, pathogen physiology, and clinical manifestations. Ann N Y Acad Sci 2007; 1105: 1–29. 17395726 10.1196/annals.1409.009

[bib4] Carvalho CL, Carvalho ILD, Zé-Zé L et al. Tularaemia: a challenging zoonosis. Comp Immunol Microb 2014; 37: 85. 10.1016/j.cimid.2014.01.002 PMC7124367 24480622

[bib5] Kingry LC, Petersen JM. Comparative review of *Francisella tularensis* and *Francisella novicida*. Front Cell Infect Mi 2014; 4: 35. 10.3389/fcimb.2014.00035 PMC3952080 24660164

[bib6] Sjodin A, Svensson K, Ohrman C et al. Genome characterisation of the genus Francisella reveals insight into similar evolutionary paths in pathogens of mammals and fish. BMC Genomics 2012; 13: 268. 22727144 10.1186/1471-2164-13-268 PMC3485624

[bib7] Lai XH, Golovliov I, Sjostedt A. Francisella tularensis induces cytopathogenicity and apoptosis in murine macrophages via a mechanism that requires intracellular bacterial multiplication. Infect Immun 2001; 69: 4691–4694. 11402018 10.1128/IAI.69.7.4691-4694.2001 PMC98551

[bib8] Xin-He Lai AS. Delineation of the molecular mechanisms of *Francisella tularensis*-induced apoptosis in murine macrophages. Infect Immun 2003; 71: 4642–4646. 12874344 10.1128/IAI.71.8.4642-4646.2003 PMC165996

[bib9] Barker JR, Klose KE. Molecular and genetic basis of pathogenesis in *Francisella tularensis*. Ann N Y Acad Sci 2007; 1105: 138–159. 17395737 10.1196/annals.1409.010

[bib10] Ancuta P, Pedron T, Girard R et al. Inability of the *Francisella tularensis* lipopolysaccharide to mimic or to antagonize the induction of cell activation by endotoxins. Infect Immun 1996; 64: 2041–2046. 8675305 10.1128/iai.64.6.2041-2046.1996 PMC174034

[bib11] Sandstrom G, Sjostedt A, Johansson T et al. Immunogenicity and toxicity of lipopolysaccharide from *Francisella tularensis* LVS. FEMS Microbiol Immunol 1992; 5: 201–210. 1419118 10.1111/j.1574-6968.1992.tb05902.x

[bib12] Baron GS, Nano FE. MglA and MglB are required for the intramacrophage growth of *Francisella novicida*. Mol Microbiol 1998; 29: 247–259. 9701818 10.1046/j.1365-2958.1998.00926.x

[bib13] Mohapatra NP, Balagopal A, Soni S et al. AcpA is a Francisella acid phosphatase that affects intramacrophage survival and virulence. Infect Immun 2007; 75: 390–396. 17060465 10.1128/IAI.01226-06 PMC1828385

[bib14] Qin A, Scott DW, Mann BJ. *Francisella tularensis* subsp. tularensis Schu S4 disulfide bond formation protein B, but not an RND-type efflux pump, is required for virulence. Infect Immun 2008; 76: 3086–3092. 18458069 10.1128/IAI.00363-08 PMC2446700

[bib15] Qin A, Zhang Y, Clark ME et al. FipB, an essential virulence factor of *Francisella tularensis* subsp. tularensis, has dual roles in disulfide bond formation. J Bacteriol 2014; 196: 3571–3581. 25092026 10.1128/JB.01359-13 PMC4187702

[bib16] Lo KY, Visram S, Vogl AW et al. Morphological analysis of *Francisella novicida* epithelial cell infections in the absence of functional FipA. Cell Tissue Res 2016; 363: 1–11. 26239909 10.1007/s00441-015-2246-0

[bib17] Wu X, Ren G, Weaver DA et al. FmvB: a *Francisella tularensis* magnesium-responsive outer membrane protein that plays a role in virulence. PLoS One 2016; 11: e0160977. 27513341 10.1371/journal.pone.0160977 PMC4981453

[bib18] Saha SS, Hashino M, Jin S et al. Contribution of methionine sulfoxide reductase B (MsrB) to *Francisella tularensis* infection in mice. FEMS Microbiol Lett 2017. 10.1093/femsle/fnw260 28108583

[bib19] Honn M, Lindgren H, Bharath GK et al. Lack of OxyR and KatG results in extreme susceptibility of *Francisella tularensis* LVS to oxidative stress and marked attenuation *in vivo*. Front Cell Infect Microbiol 2017; 7: 14. 28174696 10.3389/fcimb.2017.00014 PMC5258697

[bib20] Nano FE, Zhang N, Cowley SC et al. A *Francisella tularensis* pathogenicity island required for intramacrophage growth. J Bacteriol 2004; 186: 6430–6436. 15375123 10.1128/JB.186.19.6430-6436.2004 PMC516616

[bib21] Weiss DS, Brotcke A, Henry T et al. *In vivo* negative selection screen identifies genes required for Francisella virulence. Proc Natl Acad Sci USA 2007; 104: 6037–6042. 17389372 10.1073/pnas.0609675104 PMC1832217

[bib22] Su J, Yang J, Zhao D et al. Genome-wide identification of *Francisella tularensis* virulence determinants. Infect Immun 2007; 75: 3089–3101. 17420240 10.1128/IAI.01865-06 PMC1932872

[bib23] Kadzhaev K, Zingmark C, Golovliov I et al. Identification of genes contributing to the virulence of Francisella tularensis SCHU S4 in a mouse intradermal infection model. PLoS One 2009; 4: e5463. 19424499 10.1371/journal.pone.0005463 PMC2675058

[bib24] Kraemer PS, Mitchell A, Pelletier MR et al. Genome-wide screen in Francisella novicida for genes required for pulmonary and systemic infection in mice. Infect Immun 2009; 77: 232–244. 18955478 10.1128/IAI.00978-08 PMC2612238

[bib25] Akoh CC, Lee GC, Liaw YC et al. GDSL family of serine esterases/lipases. Prog Lipid Res 2004; 43: 534–552. 15522763 10.1016/j.plipres.2004.09.002

[bib26] Arpigny JL, Jaeger KE. Bacterial lipolytic enzymes: classification and properties. Biochem J 1999; 343 (Pt 1): 177–183. 10493927 PMC1220539

[bib27] Bornscheuer UT. Methods to increase enantioselectivity of lipases and esterases. Curr Opin Biotechnol 2002; 13: 543–547. 12482512 10.1016/s0958-1669(02)00350-6

[bib28] Tamirariel D, Rosenberg T, Navon N et al. A secreted lipolytic enzyme from Xanthomonas campestris pv. vesicatoria is expressed in planta and contributes to its virulence. Mol Plant Pathol 2012; 13: 556. 22176521 10.1111/j.1364-3703.2011.00771.x PMC6638646

[bib29] Floresdíaz M, Monturiolgross L, Naylor C et al. Bacterial Sphingomyelinases and Phospholipases as Virulence Factors. Microbiol Mol Biol Rev 2016; 80: 597. 27307578 10.1128/MMBR.00082-15 PMC4981679

[bib30] Slomiany BL, Kasinathan C, Slomiany A. Lipolytic activity of Campylobacter pylori: effect of colloidal bismuth subcitrate (De-Nol). Am J Gastroenterol 1989; 84: 1273–1277. 2801678

[bib31] Piotrowski J, Czajkowski A, Yotsumoto F et al. Sulglycotide effect on the proteolytic and lipolytic activities of Helicobacter pylori toward gastric mucus. Am J Gastroenterol 1994; 89: 232–236. 8304309

[bib32] Ruiz C, Falcocchio S, Pastor FI et al. Helicobacter pylori EstV: identification, cloning, and characterization of the first lipase isolated from an epsilon-proteobacterium. Appl Environ Microbiol 2007; 73: 2423–2431. 17293528 10.1128/AEM.02215-06 PMC1855603

[bib33] Miao EA, Miller SI. A conserved amino acid sequence directing intracellular type III secretion by Salmonella typhimurium. Proc Natl Acad Sci USA 2000; 97: 7539–7544. 10861017 10.1073/pnas.97.13.7539 PMC16581

[bib34] Ohlson MB, Fluhr K, Birmingham CL et al. SseJ deacylase activity by Salmonella enterica serovar Typhimurium promotes virulence in mice. Infect Immun 2005; 73: 6249–6259. 16177296 10.1128/IAI.73.10.6249-6259.2005 PMC1230951

[bib35] Nawabi P, Catron DM, Haldar K. Esterification of cholesterol by a type III secretion effector during intracellular Salmonella infection. Mol Microbiol 2008; 68: 173–185. 18333886 10.1111/j.1365-2958.2008.06142.x

[bib36] LaRock DL, Brzovic PS, Levin I et al. A Salmonella typhimurium-translocated glycerophospholipid:cholesterol acyltransferase promotes virulence by binding to the RhoA protein switch regions. J Biol Chem 2012; 287: 29654–29663. 22740689 10.1074/jbc.M112.363598 PMC3436183

[bib37] Lang C, Flieger A. Characterisation of *Legionella pneumophila* phospholipases and their impact on host cells. Eur J Cell Biol 2011; 90: 903–912. 21342713 10.1016/j.ejcb.2010.12.003

[bib38] Flieger A, Gongab S, Faigle M et al. Phospholipase A secreted by *Legionella pneumophila* destroys alveolar surfactant phospholipids. FEMS Microbiol Lett 2000; 188: 129–133. 10913695 10.1111/j.1574-6968.2000.tb09183.x

[bib39] Cotes K, N'Goma J, Dhouib R et al. Lipolytic enzymes in *Mycobacterium tuberculosis*. Appl Microbiol Biotechnol 2008; 78: 741–749. 18309478 10.1007/s00253-008-1397-2

[bib40] Mishra KC, de Chastellier C, Narayana Y et al. Functional role of the PE domain and immunogenicity of the Mycobacterium tuberculosis triacylglycerol hydrolase LipY. Infect Immun 2008; 76: 127–140. 17938218 10.1128/IAI.00410-07 PMC2223678

[bib41] Camus JC, Pryor MJ, Medigue C et al. Re-annotation of the genome sequence of Mycobacterium tuberculosis H37Rv. Microbiology 2002; 148 (Pt 10): 2967–2973. 12368430 10.1099/00221287-148-10-2967

[bib42] Kulp A, Kuehn MJ. Biological functions and biogenesis of secreted bacterial outer membrane vesicles. Annu Rev Microbiol 2010; 64: 163–184. 20825345 10.1146/annurev.micro.091208.073413 PMC3525469

[bib43] Brandtzaeg P, Bryn K, Kierulf P et al. Meningococcal endotoxin in lethal septic shock plasma studied by gas chromatography, mass-spectrometry, ultracentrifugation, and electron microscopy. J Clin Invest 1992; 89: 816–823. 1541674 10.1172/JCI115660 PMC442926

[bib44] Stephens DS, Edwards KM, Morris F et al. Pili and outer membrane appendages on *Neisseria meningitidis* in the cerebrospinal fluid of an infant. J Infect Dis 1982; 146: 568. 6126510 10.1093/infdis/146.4.568

[bib45] Pathirana RD, Kaparakis-Liaskos M. Bacterial membrane vesicles: biogenesis, immune regulation and pathogenesis. Cell Microbiol 2016; 18: 1518–1524. 27564529 10.1111/cmi.12658

[bib46] Horstman AL, Kuehn MJ. Enterotoxigenic *Escherichia coli* secretes active heat-labile enterotoxin via outer membrane vesicles. J Biol Chem 2000; 275: 12489–12496. 10777535 10.1074/jbc.275.17.12489 PMC4347834

[bib47] Kesty NC, Mason KM, Reedy M et al. Enterotoxigenic *Escherichia coli* vesicles target toxin delivery into mammalian cells. EMBO J 2004; 23: 4538–4549. 15549136 10.1038/sj.emboj.7600471 PMC533055

[bib48] Metruccio MM, Evans DJ, Gabriel MM et al. *Pseudomonas aeruginosa* outer membrane vesicles triggered by human mucosal fluid and lysozyme can prime host tissue surfaces for bacterial adhesion. Front Microbiol 2016; 7: 871. 27375592 10.3389/fmicb.2016.00871 PMC4891360

[bib49] Kaparakis-Liaskos M, Ferrero RL. Immune modulation by bacterial outer membrane vesicles. Nat Rev Immunol 2015; 15: 375–387. 25976515 10.1038/nri3837

[bib50] Koeppen K, Hampton TH, Jarek M et al. A novel mechanism of host-pathogen interaction through sRNA in bacterial outer membrane vesicles. PLoS Pathog 2016; 12: e1005672. 27295279 10.1371/journal.ppat.1005672 PMC4905634

[bib51] Kadurugamuwa JL, Beveridge TJ. Bacteriolytic effect of membrane vesicles from Pseudomonas aeruginosa on other bacteria including pathogens: conceptually new antibiotics. J Bacteriol 1996; 178: 2767–2774. 8631663 10.1128/jb.178.10.2767-2774.1996 PMC178010

[bib52] Altindis E, Fu Y, Mekalanos JJ. Proteomic analysis of Vibrio cholerae outer membrane vesicles. Proc Natl Acad Sci USA 2014; 111: E1548–E1556. 24706774 10.1073/pnas.1403683111 PMC3992640

[bib53] Turnbull L, Toyofuku M, Hynen AL et al. Explosive cell lysis as a mechanism for the biogenesis of bacterial membrane vesicles and biofilms. Nat Commun 2016; 7: 11220. 27075392 10.1038/ncomms11220 PMC4834629

[bib54] Elhenawy W, Bording-Jorgensen M, Valguarnera E et al. LPS remodeling triggers formation of outer membrane vesicles in Salmonella. MBio 2016; 7. 10.1128/mBio.00940-16 PMC4958258 27406567

[bib55] Roier S, Zingl FG, Cakar F et al. A novel mechanism for the biogenesis of outer membrane vesicles in Gram-negative bacteria. Nat Commun 2016; 7: 10515. 26806181 10.1038/ncomms10515 PMC4737802

[bib56] Pierson T, Matrakas D, Taylor YU et al. Proteomic characterization and functional analysis of outer membrane vesicles of Francisella novicida suggests possible role in virulence and use as a vaccine. J Proteome Res 2011; 10: 954–967. 21138299 10.1021/pr1009756

[bib57] McCaig WD, Koller A, Thanassi DG. Production of outer membrane vesicles and outer membrane tubes by *Francisella novicida*. J Bacteriol 2013; 195: 1120–1132. 23264574 10.1128/JB.02007-12 PMC3592013

[bib58] Su J, Asare R, Yang J et al. The capBCA locus is required for intracellular growth of *Francisella tularensis* LVS. Front Microbiol 2011; 2: 83. 21747799 10.3389/fmicb.2011.00083 PMC3128946

[bib59] LoVullo ED, Sherrill LA, Perez LL et al. Genetic tools for highly pathogenic *Francisella tularensis* subsp. tularensis. Microbiology 2006; 152 (Pt 11): 3425–3435. 17074911 10.1099/mic.0.29121-0

[bib60] Schmittgen TD, Livak KJ. Analyzing real-time PCR data by the comparative C(T) method. Nat Protoc 2008; 3: 1101–1108. 18546601 10.1038/nprot.2008.73

[bib61] Lawrence RC, Fryer TF, Reiter B. Rapid method for the quantitative estimation of microbial lipases. Nature 1967; 213: 1264–1265.

[bib62] Chamberlain RE. Evaluation of live tularenmia vaccine prepared in a chemically defined medium. Appl Microbiol 1965; 13: 232–235. 14325885 10.1128/am.13.2.232-235.1965 PMC1058227

[bib63] Zhang J-R, Hardham JM, Barbour AG et al. Antigenic variation in Lyme disease borreliae by promiscuous recombination of VMP-like sequence cassettes. Cell 1997; 89: 275–285. 9108482 10.1016/s0092-8674(00)80206-8

[bib64] Huntley JF, Conley PG, Hagman KE et al. Characterization of *Francisella tularensis* outer membrane proteins. J Bacteriol 2007; 189: 561–574. 17114266 10.1128/JB.01505-06 PMC1797401

[bib65] Elmi A, Nasher F, Jagatia H et al. Campylobacter jejuni outer membrane vesicle-associated proteolytic activity promotes bacterial invasion by mediating cleavage of intestinal epithelial cell E-cadherin and occludin. Cell Microbiol 2016; 18: 561–572. 26451973 10.1111/cmi.12534

[bib66] Russo BC, Horzempa J, O'Dee DM et al. A *Francisella tularensis* locus required for spermine responsiveness is necessary for virulence. Infect Immun 2011; 79: 3665–3676. 21670171 10.1128/IAI.00135-11 PMC3165480

[bib67] Chakraborty S, Monfett M, Maier TM et al. Type IV pili in *Francisella tularensis*: roles of pilF and pilT in fiber assembly, host cell adherence, and virulence. Infect Immun 2008; 76: 2852–2861. 18426883 10.1128/IAI.01726-07 PMC2446743

[bib68] Upton C, Buckley JT. A new family of lipolytic enzymes? Trends Biochem Sci 1995; 20: 178–179. 7610479 10.1016/s0968-0004(00)89002-7

[bib69] Nano FE. Identification of a heat-modifiable protein of *Francisella tularensis* and molecular cloning of the encoding gene. Microb Pathog 1988; 5: 109–119. 3237052 10.1016/0882-4010(88)90013-7

[bib70] Gray CG, Cowley SC, Cheung KK et al. The identification of five genetic loci of Francisella novicida associated with intracellular growth. FEMS Microbiol Lett 2002; 215: 53–56. 12393200 10.1111/j.1574-6968.2002.tb11369.x

[bib71] Chutkan H, Macdonald I, Manning A et al. Quantitative and qualitative preparations of bacterial outer membrane vesicles. Methods Mol Biol 2013; 966: 259–272. 23299740 10.1007/978-1-62703-245-2_16 PMC4317262

[bib72] Ogawa Y, Goda S, Morita S. The effect of Methyl-β-cyclodextrin on the differentiation of RAW264 cells into osteoclasts. Int J Oral Sci 2008; 5: 15–23.

[bib73] Galka F, Wai SN, Kusch H et al. Proteomic characterization of the whole secretome of Legionella pneumophila and functional analysis of outer membrane vesicles. Infect Immun 2008; 76: 1825–1836. 18250176 10.1128/IAI.01396-07 PMC2346698

[bib74] Ellis TN, Kuehn MJ. Virulence and immunomodulatory roles of bacterial outer membrane vesicles. Microbiol Mol Biol Rev 2010; 74: 81–94. 20197500 10.1128/MMBR.00031-09 PMC2832350

[bib75] Hynes SO, Keenan JI, Ferris JA et al. Lewis epitopes on outer membrane vesicles of relevance to Helicobacter pylori pathogenesis. Helicobacter 2005; 10: 146–156. 15810946 10.1111/j.1523-5378.2005.00302.x

[bib76] Lucas EA, Billington SJ, Carlson P et al. Phospholipase D promotes *Arcanobacterium haemolyticum* adhesion via lipid raft remodeling and host cell death following bacterial invasion. BMC Microbiol 2010; 10: 270. 20973961 10.1186/1471-2180-10-270 PMC2978216

