# Supplementary material for: Outer membrane vesicle-associated lipase FtlA enhances cellular invasion and virulence in Francisella tularensis LVS
Source: Emerg Microbes Infect. 2017 Jul 26;6(7):e66–. doi: 10.1038/emi.2017.53 (PMC5567169; doi:10.1038/emi.2017.53)
Supplement: Supplementary Information [file emi201753x1.docx]

**SUPPLEMENTARY MATERIALS**

**Title: Outer Membrane Vesicle-Associated Lipase FtlA Enhances Cellular Invasion and Virulence in *Francisella tularensis* LVS**

**Detection of the FtlA in the fractions of the bacterial culture supernatant**

Fractionation of the *F. tularensis* LVS culture supernatant was performed as described[^1^](#_ENREF_1) with modifications. Briefly, *F. tularensis* LVS were cultured in BHI broth to the late exponential phase. Bacteria were removed by centrifugation (8 000 × g, 10 min), and the supernatant was filtered using a filter with 0.45-μm pore. The resulting solution was concentrated initially with an Amicon Ultra-15 filtration unit (Merck), and used as supernatant protein (SP) fraction. The SP suspension was subjected to ultracentrifugation at 100 000 × g for 1 h and the supernatant was collected and used as soluble protein supernatant (SSP) fraction without OMVs. Proteins in both SP and SSP fractions were precipitated overnight with ice-cold trichloroacetic acid at a final concentration of 10% and collected by centrifugation at 8 000 × g for 1 h at 4°C. The resulting pellets were washed five times with 95% ethanol and then dried at room temperature. The pelleted proteins were dissolved in solution to the 1/250 of the initial volume of SP and SSP. Proteins was separated by SDS-PAGE and probed with the antiserum against FtlA.

**Substrate specificity of OMV-associated FtlA**

The *p*-nitrophenyl esters with carbon chain lengths ranging from C_4_ to C_18_ were purchased from Sigma-Aldrich and used for as substrate specificity assay as described.[^2^](#_ENREF_2) Briefly, *p*-nitrophenyl butyrate (C_4_) and *p*-nitrophenyl decanoate (C_10_) were dissolved in acetonitrile, and the *p*-nitrophenyl laurate (C_12_), *p*-nitrophenyl myristate (C_14_), *p*-nitrophenyl palmitate (C_16_), or *p*-nitrophenyl stearate (C_18_) were dissolved in a mixture of acetonitrile and 2-propanol (1:4) to the concentration of the 100 mM. Outer membrane vesicles (OMVs) freshly purified from *F. tularensis* LVS or isogenic Δ*ftlA* mutant were diluted with lipase buffer (1.25 mM NaH_2_PO_4_, 3.75 mM Na_2_HPO_4_, 0.05 % CaCl_2_, 4.72 mM sodium deoxycholate sodium, 0.9 % gum Arabic, pH 8.0) to a concentration of 50 μg/ml. For lipolytic assay, 10 μl of the dissolved substrate was added to 990 μl of suspension, and the mixtures were incubated at 37 °C for 1 h. The reactions were terminated by placing in an ice bath and absorbance of each sample was recorded at 410 nm using a 96-well plate reader (Multiscan MK3, Thermo Scientific). Substrates without OMVs were used as blank reaction control.

**REFERENCE**

1. Galka F, Wai SN, Kusch H, Engelmann S, Hecker M, Schmeck B*, et al.* Proteomic characterization of the whole secretome of Legionella pneumophila and functional analysis of outer membrane vesicles. *Infect Immun* 2008; **76**(5)**:** 1825-1836.

2. Prive F, Kaderbhai NN, Girdwood S, Worgan HJ, Pinloche E, Scollan ND*, et al.* Identification and characterization of three novel lipases belonging to families II and V from Anaerovibrio lipolyticus 5ST. *PLoS One* 2013; **8**(8)**:** e69076.
