# Supplementary material for: Outer membrane vesicle-associated lipase FtlA enhances cellular invasion and virulence in Francisella tularensis LVS
Source: Emerg Microbes Infect. 2017 Jul 26;6(7):e66–. doi: 10.1038/emi.2017.53 (PMC5567169; doi:10.1038/emi.2017.53)
Supplement: Supplementary Table S1 [file emi201753x2.pdf]

Table S1. Primers used in this study

Primer name	Primer sequence (5'-3')
Pr1651	CGCGGATCCAATATGCGACAAATAAATAGACT
Pr1652	CCGCTCGAGTCATATATCAAGCTTCCATAAATCA
Pr2000	GCGGTACCATGCGACAAATAAATAGACTGGT
Pr2001	GCGGATCCTCATATATCAAGCTTCCATAAATCA
Pr2002	GCGGTACCATGCGACAAATAAATAGACTGGTAGTTTTTGGAGATGCTCTTTTAG
Pr2003	GCGGATCCTCATATATCAAGCTTCCATAAATCA
Pr2004	GGATTTGCTGTAGGAGTGCGTAAC
Pr2005	ATCAGCAAACGCAAAAGTATCACC
Pr2006	GGGGTACCATGAGTGAGATGATAACAAGACAACA
Pr2007	CGGGATCCTGCAGCTGCAATATATCCTA
Pr2008	GCGGTACCATGGGTTCAGATAATATCGATACGTCA
Pr2009	GCGGATCCTTAGTTAGCTTCTTTAAGTGGAGCTG
Pr1089	CTTGTCTCGAGTATGCGGATATTCGTGAGC
Pr1090	ATGACAATAATCCCAACACTACCAGTCTATTTATTTGTCG
Pr1091	CGACAAATAAATAGACTGGTAGTGTTGGGATTATTGTCAT
Pr1092	CTTGTCTCGAGTTAGCTGGAGTAGAGTTTATTCA
Pr1601	AAGGATTACATATGCGACAAATAAATAGA
Pr1602	TAGCTTCATATGAAAACTCCTTTAAATACGCTGCGCTATTAAATCTTGAC

**Cotranscription**	
Pr2030 (FTL_0427-FTL_0428)	TCGAGGCAAAAGATTTGGCAC
Pr2031 (FTL_0427-FTL_0428)	CTGACCAACCTTGCTTGCCT
Pr2032 (FTL_0428-FTL_0429)	GCGTAAAGCTGAAATTTTAGATAGTAC
Pr2033 (FTL_0428-FTL_0429)	TCCAAGCAAGATTATCAAAAGCTGT
Pr2034 (FTL_0429-FTL_0430)	TCTTAGGAATACAAGCCCACCC
Pr2035 (FTL_0429-FTL_0430)	TTGTCCCATGTGTCAACGCT
Pr2036 (FTL_0430-FTL_0431)	TGAAAATGTTACCGATGCCTGT

Pr2037 (FTL_0430-FTL_0431)	TGCCAACTATGTTCAGCGCT
**qPCR**	
Pr2020 (FTL_0427)	GCCTGACGCTTATATGTGGATG
Pr2021 (FTL_0427)	TCACGCACGTTTTTCTCGTC
Pr2022 (FTL_0428)	AAACGAACAGGCAAGCAAGG
Pr2023 (FTL_0428)	ATGTCTTACGCGGCTGATTG
Pr2024 (FTL_0429)	AGGGTTGAACGAGGTCCTAAAG
Pr2025 (FTL_0429)	GAAATGGCTCCATCCATGGC
Pr2026 (FTL_0431)	TAGCGCTGAACATAGTTGGC
Pr2027 (FTL_0431)	CTAAACTGACAGTGGCATACCC
Pr2028 (16S rRNA)	ACCGATACTGACACTGA

Pr2029 (16S rRNA)	TTACACCGACTCCAACA
