# Supplementary material for: Outer membrane vesicle-associated lipase FtlA enhances cellular invasion and virulence in Francisella tularensis LVS
Source: Emerg Microbes Infect. 2017 Jul 26;6(7):e66–. doi: 10.1038/emi.2017.53 (PMC5567169; doi:10.1038/emi.2017.53)
Supplement: Supplementary Figure S1 [file emi201753x3.pdf]

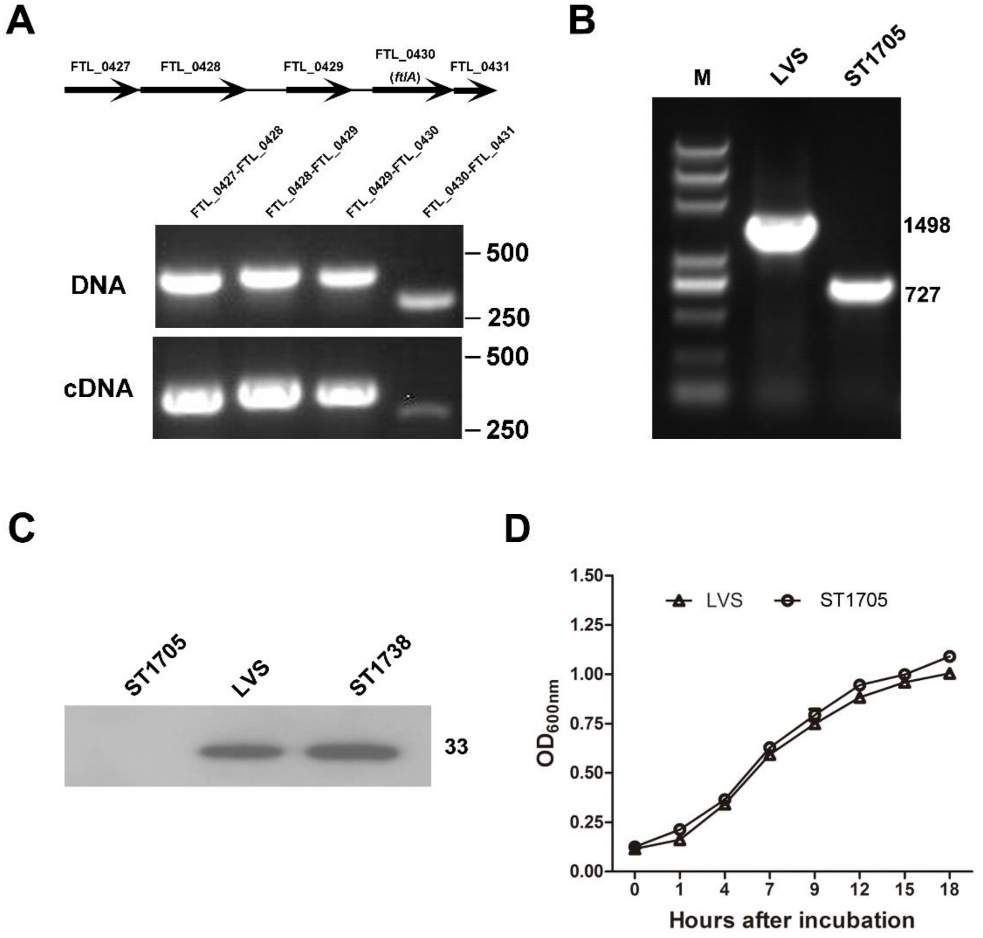

Figure S1. Construction and confirmation of *F. tularensis* LVS *ftlA* deletion mutant. (A) Co-transcription of *ftlA* locus genes determined by RT-PCR. FTL_0427-FTL_0428, FTL_0428-FTL_0429, FTL_0429-FTL_0430, and FTL_0430-FTL_0431 junctions were amplified with DNA or cDNA as a template. The sizes of DNA were indicated at the right in bp. (B) The *ftlA* deletion mutant ST1705 was examined for the loss of the *ftlA* gene by PCR using primer pair Pr2004/Pr2005 (Table S1). LVS represents the wild type strain. The sizes of DNA were indicated at the right in bp. (C) The expression of the FtlA was detected by Western blotting with polyclonal antibody against the recombinant FtlA. The sizes of proteins were indicated at the right in kDa. (D) LVS and ST1705 were cultured individually in the MHB. Optical density values were

determined for each culture at the indicated time-points. The values represent means ± SD of triplicate samples.
