# Supplementary material for: Outer membrane vesicle-associated lipase FtlA enhances cellular invasion and virulence in Francisella tularensis LVS
Source: Emerg Microbes Infect. 2017 Jul 26;6(7):e66–. doi: 10.1038/emi.2017.53 (PMC5567169; doi:10.1038/emi.2017.53)
Supplement: Supplementary Figure S2 [file emi201753x4.pdf]

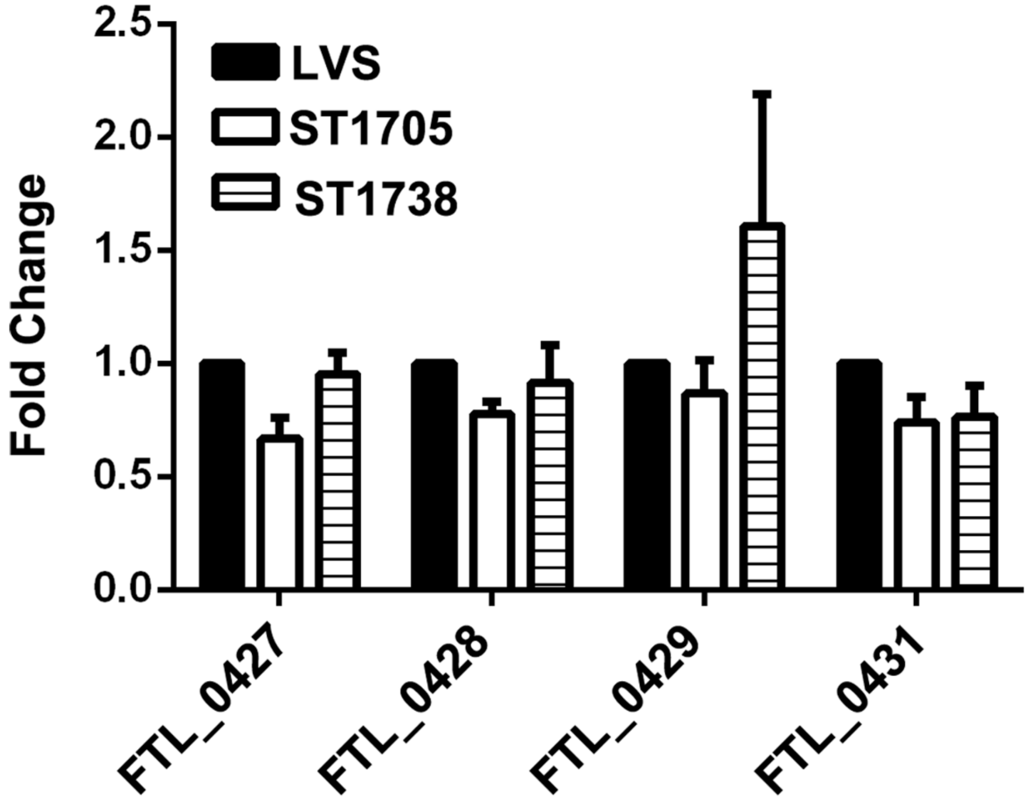

Figure S2. Transcription of *ftlA* locus genes in wild-type LVS, the isogenic derivative ST1705 and the *in trans* complemented ST1738. All reactions were performed in triplicate with three independent RNA preparations. Data collection was performed with the relative transcription level of each gene normalized to 16S rRNA gene. Results were shown as the mean fold changes versus LVS ± SE. Statistical significance was determined by Student *t* test.
