# Supplementary material for: Outer membrane vesicle-associated lipase FtlA enhances cellular invasion and virulence in Francisella tularensis LVS
Source: Emerg Microbes Infect. 2017 Jul 26;6(7):e66–. doi: 10.1038/emi.2017.53 (PMC5567169; doi:10.1038/emi.2017.53)
Supplement: Supplementary Figure S3 [file emi201753x5.pdf]

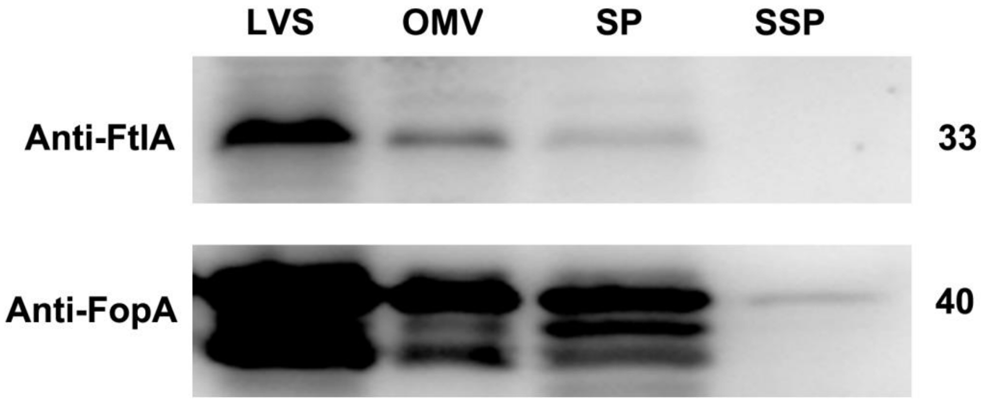

Figure S3. Detection of OMV-associated FtlA from *F. tularensis* LVS.

The proteins from *F. tularensis* LVS supernatant containing OMV (SP) or not (SSP) were pelleted using TCA. These proteins were detected with specific antiboby against FtlA and FopA. The whole cell lysate (LVS) and OMV from *F. tularensis* LVS were used as control. The sizes of proteins were indicated at the right in kDa.
