# Supplementary material for: Outer membrane vesicle-associated lipase FtlA enhances cellular invasion and virulence in Francisella tularensis LVS
Source: Emerg Microbes Infect. 2017 Jul 26;6(7):e66–. doi: 10.1038/emi.2017.53 (PMC5567169; doi:10.1038/emi.2017.53)
Supplement: Supplementary Figure S4 [file emi201753x6.pdf]

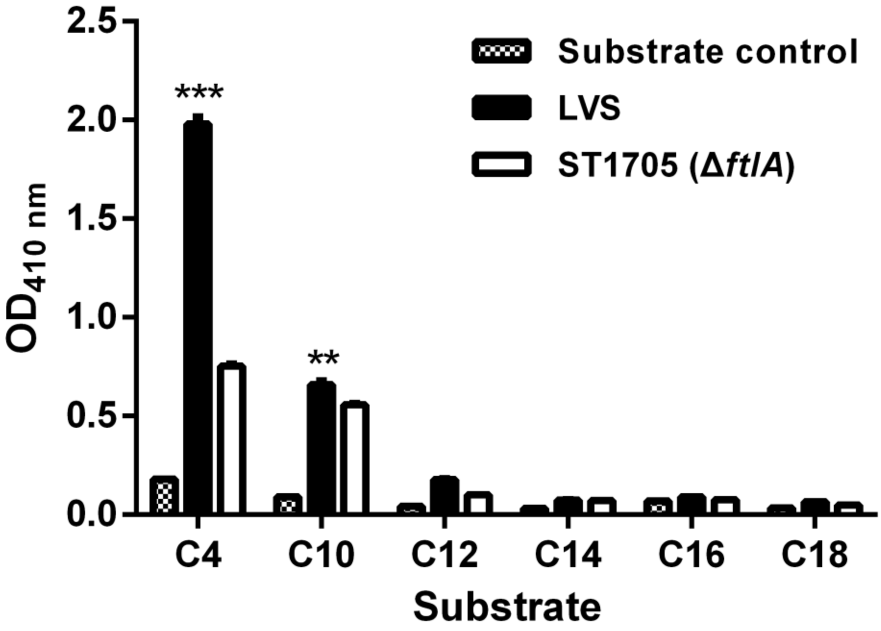

Figure S4. Substrates hydrolysis of OMV-associated FtlA.

Density-gradient purified OMVs of wild-type LVS and Δ*ftlA* mutant were separately added to lipase assay buffer containing *p*-nitrophenyl ester substrates with C_4-C_{18} acyl chains, incubated at 37 °C for 1 h and transferred to an ice bath to terminate the reaction. The absorbance of each sample was detected at 410 nm with clear 96 well plates. The OMV-associated FtlA displayed preference for esters with short acyl chains. The values represent means ± SD of triplicate samples. *P* values were calculated using an unpaired *t*-test, comparing ST1705 with the wild type LVS.
